# Integrating Audiovisual Data for Short-Term Particulate Matter Concentration Prediction on Urban Sidewalks

**DOI:** 10.1021/acs.est.6c03195

**Published:** 2026-07-22

**Authors:** Xujing Yu, Jun Ma, Waishan Qiu, Wei He, Feifeng Jiang

**Affiliations:** † Department of Urban Planning and Design, Faculty of Architecture, 366136 The University of Hong Kong, Hong Kong 000000, China; ‡ State Key Laboratory of Information Engineering in Surveying, Mapping and Remote Sensing, 544334 Wuhan University, Wuhan 430072, China; § Department of Geography, 594642 Hong Kong Baptist University, Hong Kong 000000, China

**Keywords:** short-term air pollution prediction, audiovisual data, sidewalk-level particle matter concentrations, machine learning

## Abstract

Urban air pollution severely impacts pedestrians on sidewalks, yet traditional fixed-site monitoring lacks the spatial coverage needed for fine-scale exposure assessment due to high costs. This study proposes a novel machine-learning framework to predict short-term sidewalk PM_2.5_ and PM_1_ concentrations using multimodal audiovisual features extracted from self-collected street-view videos, alongside meteorological and background pollution data. Based on a mobile monitoring campaign in Shenzhen, China, we evaluated multiple models (linear regression, XGBoost, and LightGBM) across different temporal resolutions (10 s and 1 min) and validation strategies. LightGBM achieved the best performance, yielding R^2^ values of 0.64–0.65 for 10 s predictions and 0.80 for 1 min predictions under random cross-validation. Under rigorous spatial cross-validation, the model maintained moderate generalizability, with R^2^ reaching 0.41–0.48 at the 1 min resolution. Furthermore, developing a hybrid model that incorporated static geospatial context further improved the overall predictive accuracy. Variable interpretation revealed that while background PM and meteorology were dominant predictors, dynamic audio-derived features and visual indicators provided substantial additional predictive power. These findings demonstrate that integrating multimodal audiovisual sensing with ancillary data enables scalable, high-resolution estimation of street-level PM, effectively complementing conventional monitoring for urban air-quality management.

## Graphical abstract

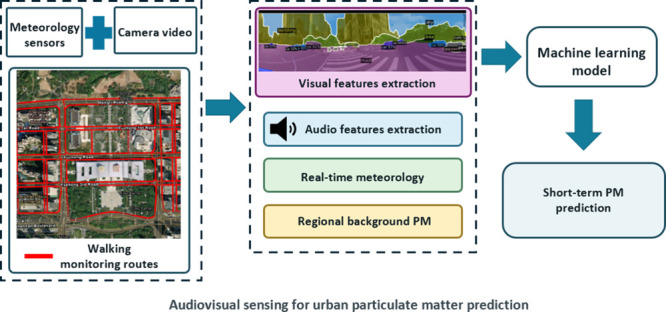

## 1 Introduction

Air pollution has emerged as one of the most critical public health challenges worldwide, with adverse effects on urban environments and human health.^1^ Road traffic emissions are widely acknowledged as a major contributor to urban air pollution.^2,3^ Pedestrian-level air pollution is particularly concerning, as individuals walking along sidewalks are in close proximity to traffic emissions, which heightens their exposure and associated health risks.^4^ To monitor urban air pollution, governments and environmental authorities worldwide have established networks of fixed-site monitoring stations, including roadside sensors, to measure the air quality. These monitoring stations provide highly accurate pollutant-concentration data. However, because of their high installation and maintenance costs, such networks are often sparsely distributed, resulting in limited spatial coverage. Given that air pollutant concentrations can vary significantly over small distances and brief time intervals, sparse and static monitoring networks cannot fully capture the spatiotemporal dynamics of road-level air pollution.^5^ With advancements in air pollution sensing technology, numerous studies have explored the use of mobile monitoring equipment for road air pollution assessment.^6^ However, mobile monitoring methods generally require substantial resources, including costly equipment.^7^ Even low-cost sensors remain inaccessible to many and necessitate frequent calibration to ensure long-term data reliability.^8^

To reduce monitoring costs, several predictive models have been developed, including numerical simulations^9^ and statistical regression models.^10^ Numerical simulations can provide high-resolution air pollution predictions but are computationally intensive and time-consuming, particularly for large-scale modeling.^11^ Statistical regression models, encompassing both traditional regression approaches and machine learning techniques, frequently use geographical predictor variables to estimate air pollution levels.^12,13^ Geographical predictor variables are useful for capturing the spatial variance in the impacts of natural and built environments on air pollution. However, they are typically derived from simplified geodatabases that cannot fully reflect the complexity of real urban environments. Moreover, the infrequent updates to geographic databases mean that these variables only capture the static characteristics of cities, limiting their ability to predict short-term changes in air pollution. Given the dynamic nature of urban traffic and environmental conditions, accurately predicting short-term variations in road air pollution is essential. Meteorological data,^14^ vehicle count,^15^ and vehicular emissions^16^ have been identified as critical variables for predicting short-term street-level air pollution in previous studies. These dynamic predictors reflect ongoing changes in urban environments and traffic conditions. In recent years, the rapid advancement of computer vision technology has enabled researchers to incorporate image-based data into air pollution forecasting. Some studies have employed static street view images to predict air quality,^17,18^ while others have utilized dynamic video data to estimate short-term air pollution fluctuations.^7,19^

Nevertheless, most of these studies rely solely on visual information from videos, while audio data are often overlooked. This reliance on visual data may limit the ability to fully understand certain environmental aspects, particularly those that are better captured through sound. For example, when vehicles are ignited on the road, a significant number of particles are emitted, but this detail is often imperceptible in images alone. Incorporating audio data can enhance the ability to more accurately forecast air pollution exposure. Furthermore, while images can be used to monitor traffic conditions, they often capture sensitive information, such as pedestrian faces and license plates, raising privacy concerns.^20^ Compared to images, urban sound data are less privacy-invasive. Previous studies have investigated the relationship between local noise pollution and traffic-related air pollution.^21^ Hong et al. used both image and audio data as inputs for CNN models to estimate UFP parameters and concentrations alongside other variables.^22^ However, audio data, despite their potential, remain underutilized in air pollution research. In addition, most mobile monitoring studies have relied on vehicles as the primary platform for air pollution measurement. Although vehicle-mounted sensors are effective for large-scale monitoring of urban road air pollution, they are limited to specific routes and fail to measure pollution levels on the sidewalks. As pedestrians are among the most vulnerable groups to the effects of air pollution, it is critical to develop methods for monitoring air pollution on urban sidewalks to better protect human health.

To address these gaps, this study proposes a novel framework for predicting short-term particulate matter (PM) concentrations on urban sidewalks using audiovisual features extracted from readily available camera footage. We conducted an air pollution monitoring campaign on urban sidewalks in the central business district (CBD) of Shenzhen, China, measuring PM_1_ and PM_2.5_ concentrations while simultaneously collecting real-time video footage. Using computer vision and audio processing techniques, we extracted audiovisual features from the videos and employed several machine-learning models to predict short-term PM concentration levels.This work demonstrates the potential of multimodal, widely obtainable sensing data to complement traditional monitoring and support higher resolution assessments of short-term pedestrian exposure to particulate pollution.

## 2 Materials and Methods

### 2.1 Study Area and Data Collection

This study focused on the central business district (CBD) of Shenzhen, a prominent megacity in Guangdong Province, southern China. CBD serves as the financial hub of Shenzhen, hosting key landmarks such as the Shenzhen Stock Exchange, numerous high-end shopping centers, and municipal cultural facilities. Additionally, the northern part of the area features a large green space, the Lianhuashan Park. Characterized by high-rise buildings, a complex urban built environment, and significant pedestrian and vehicular traffic, the area presents an ideal setting for studying the pedestrian exposure to air pollution. To systematically assess air quality, monitoring routes were meticulously designed (shown in Figure 1) to cover sidewalks on both sides of major and minor roads within the study area. The entire route spans 31.391 km.

**Figure 1:**
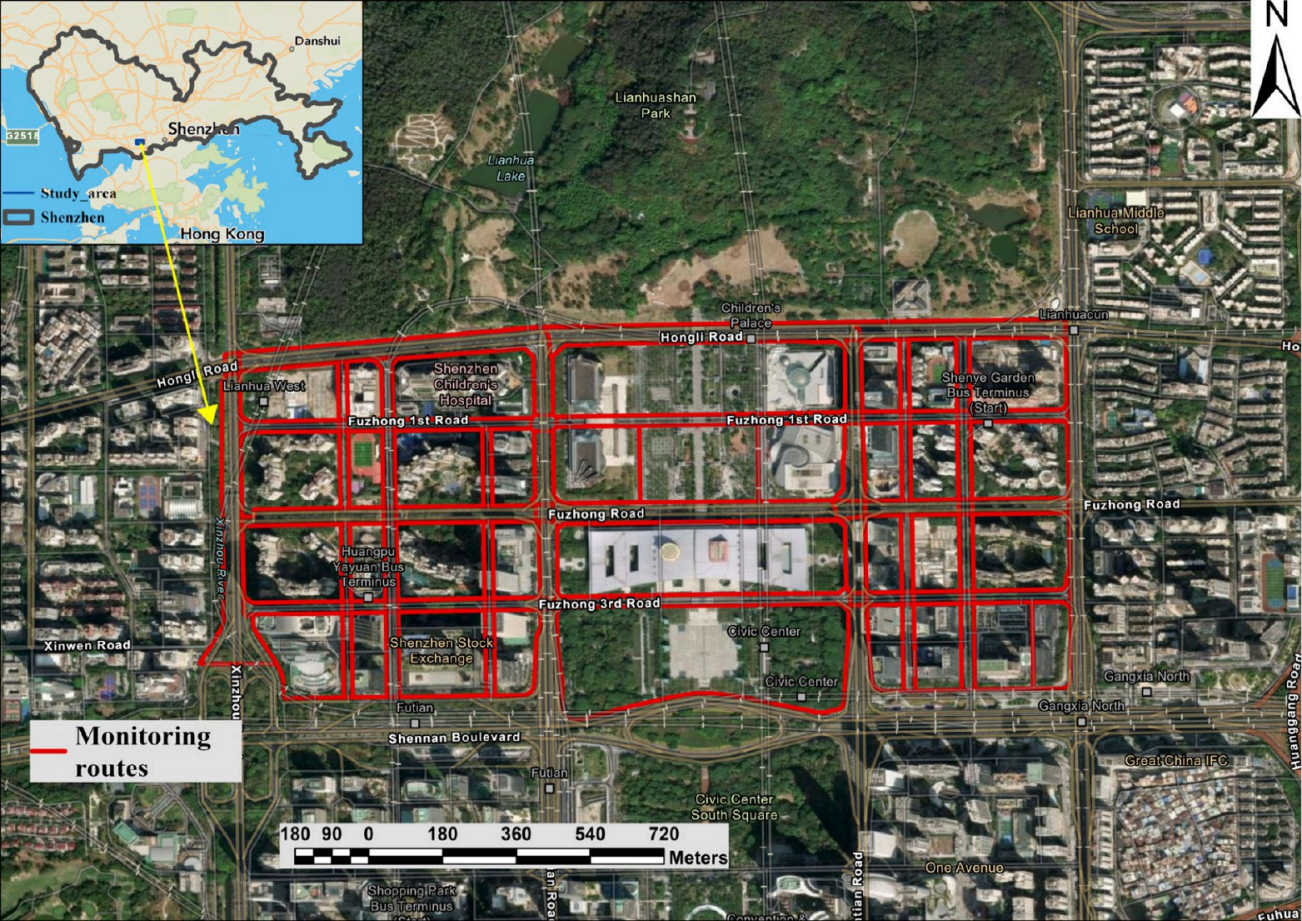
Monitoring routes in the CBD of Shenzhen.

Data collection was conducted from July 1 to July 8, 2024, under sunny-weather conditions. Four research assistants were recruited to walk the monitoring routes repeatedly each day to collect the air pollution data. Prior to the campaign, comprehensive training was provided to ensure familiarity with the monitoring routes and proper use of instruments. For this study, the portable air quality monitoring device, the AirBeam3 (https://www.habitatmap.org/airbeam, HabitatMap, United States of America), was employed to monitor PM_1_ and PM_2.5_ concentrations. The AirBeam, widely utilized in previous mobile air pollution monitoring studies,^23,24^ has demonstrated efficiency and reliability in accurately capturing air pollution data.

As shown in Figure 2(a), the AirBeam3 was attached to the research assistants’ necks using a lanyard. The device was oriented vertically with the intake vent facing downward and ensured to be unobstructed by clothing, consistent with the manufacturer’s guidelines for mobile monitoring. The device integrates a Plantower PMS7003 sensor for measuring PM concentrations and includes sensors for relative humidity, temperature, and geolocation via GPS to collect meteorological and location data (Figure 2(b)). Simultaneously, an Insta360 ONE RS panoramic camera was mounted on each assistant’s helmet to record street-view videos throughout the campaign. To minimize interperson variability that could affect measurements (e.g., due to walking style or body posture), all research assistants received comprehensive training prior to the campaign. This training emphasized maintaining a normal walking speed, keeping the device stable and exposed, and adhering to the predefined routes.

**Figure 2:**
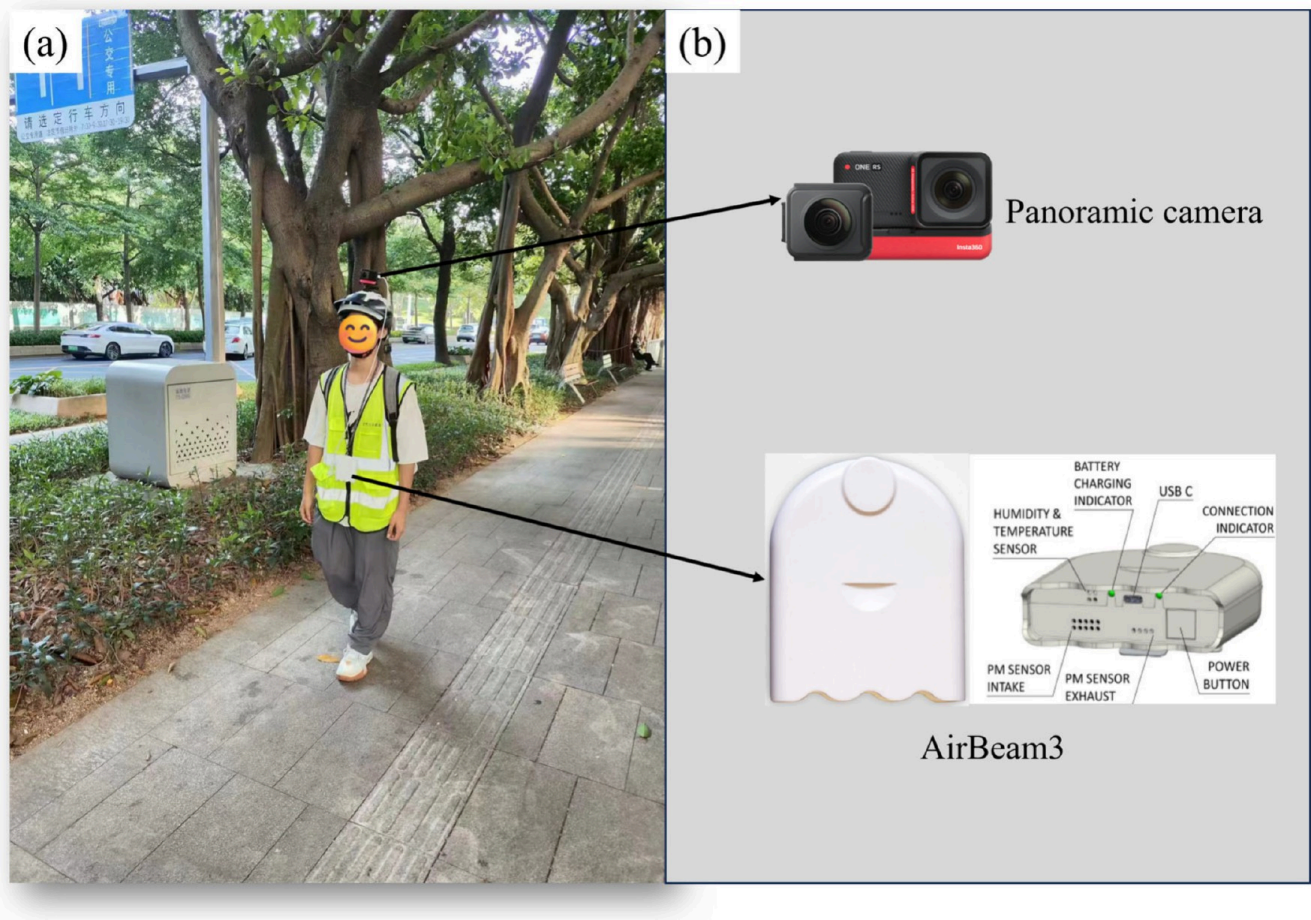
Photos about: (a) the method and process for monitoring; (b) the main devices used in the campaign.

The concentration of PM (μg/m^3^) was collected at a 5 s resolution, along with temperature (°F), relative humidity (%), and GPS information (longitude and latitude). Data collection took place during peak commuting hours—specifically from 7:30 to 9:30 a.m. and 5:30 to 7:30 p.m.—to capture pollution levels under conditions of high pedestrian and vehicular activity. After data cleaning, over 107,000 valid data points were retained. To ensure accuracy, all pollution data points were calibrated. The calibration process began with a premonitoring device calibration, wherein PM measurements from the AirBeam3 devices were compared against those from a regulatory-grade instrument, Profiler Model 212.^25^ During this calibration period, eight linear regression formulas were developed (four devices and two pollutant types) for calibration purposes. These formulas were then applied to the pollution data points collected during the monitoring campaign. As detailed in Supporting Information Section 1, the calibration demonstrated a strong linear relationship (R^2^ > 90%) with the regulatory-grade monitor. Given the duration of our monitoring campaign, this rigorous calibration provides strong evidence of high accuracy and precision within the scope of this study.

In addition to PM data, temperature and relative humidity were also measured using AirBeam3 devices. The built-in meteorology sensors in the AirBeam3 devices are Texas Instruments HDC1080DMBR sensors (https://www.ti.com/sensors/humidity-sensors/overview.html). Audiovisual variables were extracted from videos recorded by four panoramic cameras, yielding approximately 50 h of video data corresponding to the air pollution measurements.

### 2.2 Video Data Processing

#### 2.2.1 Visual Variables Extracted from Images

During the air pollution mobile monitoring campaign, videos were recorded to capture the surrounding environment. To reduce image redundancy and computational load, video frames were extracted at a constant rate of one frame every 5 s. Each image had a resolution of 1280 × 640 pixels. Subsequently, an image panoptic segmentation technique was applied to detect traffic-related objects and classify the pixels within each image, shown in Figure 3. The panoptic segmentation model employed in this study was the Panoptic Feature Pyramid Network, implemented using Detectron2, a widely used AI library for computer vision tasks developed by Facebook.^26^ The model achieved a box average precision of 42.4% and a mask average precision of 38.5% on the COCO data set. We used a pretrained version of the model to perform inference on our self-collected video frames. Finally, traffic-related object counts and the area proportions of pixels occupied by specific visual elements of the urban environment were calculated. In this study, the detected object variables included pedestrians, cars, bicycles, buses, and trucks. The segmented pixel area variables included sky, buildings, trees, grass, roads, pavements, cars, buses, and trucks.

**Figure 3:**
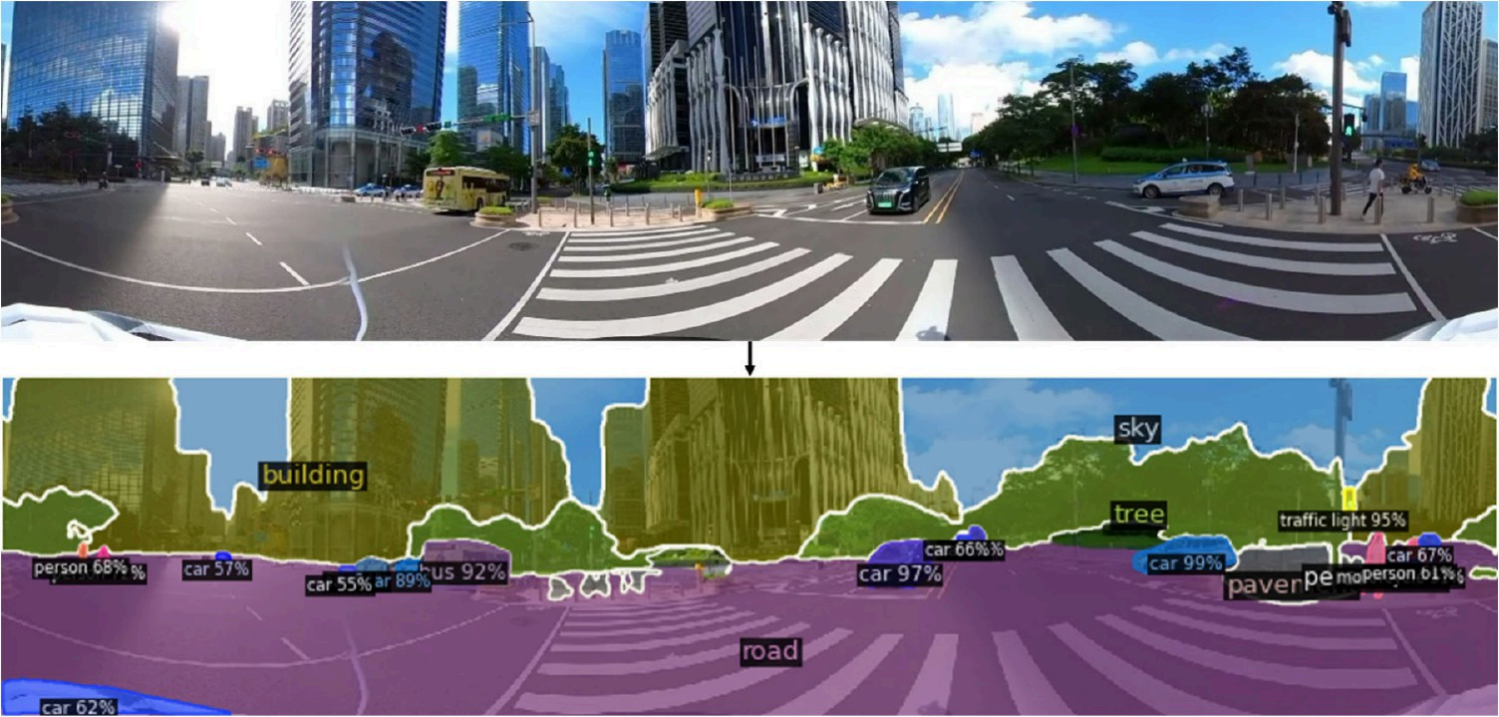
Use of the panoptic segmentation for objects detection and pixels classification in the self-collected image.

#### 2.2.2 Audio Variables Extracted from Audio Clips

Audio variables were extracted from the self-collected videos by dividing them into continuous audio clips, each lasting 10 s. These audio clips were saved in the .WAV format with a sampling rate of 44.1 kHz. To analyze the audio information, a Python toolkit called *scikit-maad*,^27^ which facilitates the quantitative analysis of audio recordings, was employed. The toolkit was used to calculate basic indicators, such as sound pressure level (SPL), which reflects sound intensity, as well as other acoustic soundscape indices, such as the acoustic complexity index (ACI) and time-frequency derivation index (TFSD).

Although general soundscapes can be calculated using existing tools, there are no specialized indicators or tools designed for analyzing the soundscape of urban traffic or sidewalks. To capture the unique soundscape characteristics of the sampling route, we referred to previous research^28–30^ and utilized an advanced deep learning model to classify soundscape types on urban sidewalks. Using a transfer learning approach, we fine-tuned the Audio Spectrogram Transformer (AST) model^31^ to classify audio into seven soundscape classes: car passing, car engine, car horn, construction, pedestrians, animals, and ambient sound. Details regarding the fine-tuning process and the performance of the AST model are provided in Supporting Information Section 2. After fine-tuning, the AST model was used to predict the probability of the presence of the seven soundscapes in the self-collected audio clips. Descriptions of the selected audiovisual predictor variables are also available in Table S2.

### 2.3 General Predictor Variables

In addition to the audiovisual features extracted from videos, three additional categories of predictor variables were incorporated into the models: background air pollution concentrations, meteorological data, and geospatial variables. Roadside air pollution concentrations are influenced not only by local emission sources (e.g., nearby traffic) but also by regional background pollution that is advected from surrounding areas. To account for this regional contribution, we included hourly background PM_2.5_ concentrations measured at government-operated fixed-site monitoring stations in Shenzhen. Because PM_1_ is a major component of PM_2.5_, hourly fixed-site PM_2.5_ measurements were also used as proxies for background PM_1_ levels. Meteorological conditions play a critical role in the dispersion and accumulation of air pollutants. Temperature and relative humidity were measured in real-time by the AirBeam3 devices during the monitoring campaign. These measurements were synchronized with the PM data and averaged over the same 10 s intervals to serve as dynamic meteorological predictors.

While the audiovisual features capture the dynamic, real-time conditions at each monitoring location, they may not fully account for the static spatial context that may influence local air pollution levels. To complement the audiovisual data and enable the model to learn the influence of persistent geospatial factors, we constructed a hybrid model that integrates both dynamic audiovisual features and static geospatial variables. Geospatial variables characterize the built environment and land use surrounding each monitoring location. Based on a comprehensive review of previous studies investigating the relationship between urban form and air pollution,^32–34^ we focused on three categories of geospatial features: building morphology, Points of Interest (POIs), and land cover. Building morphology influences local airflow patterns and pollutant dispersion.^35^ POIs serve as proxies for anthropogenic activity intensity. For example, road furniture POIs (e.g., traffic signals) are associated with vehicle idling and acceleration, which may elevate local pollution levels.^36^ The Normalized Difference Vegetation Index (NDVI) represents vegetation cover, which can have complex effects on air pollution. Building morphology and POI data were obtained from AMAP (www.amap.com), while green space was represented using the NDVI, calculated from Sentinel-2 Level-2A Surface Reflectance imagery accessed via Google Earth Engine (GEE; collection: COPERNICUS/S2_SR). Images acquired between March and September 2023 were filtered to the study area with a cloud pixel percentage threshold of less than 10% to ensure image quality. A mosaic composite was generated from the filtered collection, and NDVI was computed at 10 m spatial resolution using the standard formula: NDVI = (B8 – B4)/(B8 + B4), corresponding to the near-infrared (B8) and red (B4) bands. Details of the geospatial variables are summarized in Table S3. Given the large number of potential geospatial variables that could be formulated, we employed Recursive Feature Elimination (RFE) to select the most informative subset for model inclusion. RFE is an iterative feature selection method that recursively removes the least important features based on model coefficient weights or feature importance scores, retaining an optimal set of predictors that maximizes model performance. The detailed implementation of RFE is also described in Supporting Information Section 4.

### 2.4 Model Fitting

#### 2.4.1 Data Aggregation

In this study, air pollution data collection was synchronized with video recording to estimate short-term variations in air pollution concentrations. The study unit was defined as the average of PM concentrations measured over 10 s, aligning with the duration of each audio clip. Images and meteorological data were also matched to each audio clip by calculating their average values over the same 10 s intervals. To further assess the model’s performance at time scales more stable for health exposure assessments, we also aggregated the 10 s data into 1 min averages by calculating the mean of all 10 s measurements within each consecutive 60 s window along the monitoring routes. Because the audiovisual model extracted features directly from street-level imagery and audio, the spatial dimension naturally varied and followed the actual moving trajectory. Consequently, each 1 min chunk represented the continuous sequence of microenvironments experienced along that specific meandering path, rather than a fixed spatial area. To comprehensively understand the effect of temporal scales on model performance, we further conducted a sensitivity analysis by aggregating the data into intervals up to 10 min. The detailed results are presented in Supporting Information Section 5. For geospatial data in the hybrid model, the temporal study units were linked to built environment and land-use data based on their latitude and longitude. To analyze the effects of geospatial variables at finer scales, a buffer-based feature augmentation approach was applied,^33^ with buffer radii ranging from 100 to 500 m (100 m, 200 m, 300 m, 400 m, 500 m).

#### 2.4.2 Regression Models and High-Impact Variable Identification

In this study, several advanced machine learning techniques were employed to develop regression models capable of accurately predicting air pollution levels. Two widely used machine learning models—XGBoost^37^ and LightGBM^38^ —were selected. These advanced tree-based ensemble models were specifically chosen for their state-of-the-art performance on complex, heterogeneous environmental data.^19,39^ They are particularly well-suited for handling our multimodal data set, which integrates visual, audio, meteorological, and geospatial features with vastly different scales and distributions. Furthermore, XGBoost offers robust regularization (L1 and L2) to prevent overfitting, while LightGBM utilizes histogram-based algorithms and gradient-based one-side sampling, making it exceptionally efficient in training speed and memory consumption for large-scale environmental data sets. Additionally, linear regression was employed for comparison to baseline the nonlinear predictive capabilities of the ensemble models. The selection of model training hyperparameters was automated using the Automated Machine Learning (AutoML) framework.^40,33^ Beyond predicting PM concentrations, the study also focused on identifying high-impact variables contributing to the model’s predictions. To achieve this, SHapley Additive exPlanations (SHAP)^41^ were utilized. SHAP values provide a consistent and interpretable framework for quantifying the contribution of each feature to individual predictions. By aggregating SHAP values across all samples, the study ranked the importance of input variables, revealing the key factors influencing PM concentration levels.

#### 2.4.3 Evaluation Metrics and Validation Strategies

Model performance was evaluated using two key metrics: the coefficient of determination (R^2^) and mean squared error (MSE). R^2^ measured the proportion of variance in PM concentrations explained by the model, while MSE quantified the average squared difference between predicted and observed values, highlighting the model’s ability to minimize prediction errors. Higher R^2^ values and lower MSE values indicate better-performing models. To rigorously assess the predictive capability and generalizability of our models, we employed three distinct validation strategies that account for different sources of variability and potential overfitting:

(1) Random k-fold cross-validation (Random CV): The data set was randomly divided into five equal-sized folds. Each fold was used once as a validation set, while the remaining four folds were used for training. This process was repeated five times, and the results were averaged. This standard approach provides a baseline estimate of the model performance. It was implemented using the *cross_val_score* function from the Python *scikit-learn* package.
(2) Monte Carlo cross-validation (random subsampling): To assess the stability of model performance across different random data splits, we performed Monte Carlo CV with 50 iterations. In each iteration, 80% of the data were randomly selected for training, and the remaining 20% was held out for validation. The mean and variance of the performance metrics across all of the iterations were then calculated. This method, implemented using the *ShuffleSplit* function from *scikit-learn*, provides insights into the robustness of the model’s predictive capability.
(3) Spatial cross-validation: To rigorously test the model’s ability to generalize to unseen geographic areas and to account for spatial autocorrelation, we employed two distinct spatial blocking approaches:
  (a) Spatial route-based CV: The study area was partitioned into three large geographic zones on the basis of mobile monitoring routes. In each iteration, data from two zones were used for training and the model was validated on the held-out zone. This process was repeated three times with each zone serving as the validation set once. This strategy provides a highly conservative estimate of model performance when extrapolating to entirely new, large-scale macroregions.
  (b) Spatial grid-based CV: To ensure a more granular and sufficiently random spatial partition, the study area was divided into 100 × 100 m grids, resulting in 204 distinct valid spatial zones. The 10 s pollution measurements and their associated predictors were spatially allocated to these grids. A 5-fold cross-validation was then applied, where in each fold, 80% of the grids were used for training and the remaining 20% were held out for testing. This grid-based strategy provides a robust and realistic evaluation of the model’s spatial generalizability across finely resolved urban microenvironments.

For most validation strategies, we evaluated model performance at two temporal resolutions: the 10 s aggregation and the 1 min averages. However, the spatial grid-based CV was exclusively evaluated at the 10 s resolution because a 1 min moving window would cover a distance that frequently spans across multiple grids, leading to ambiguous spatial allocation and an insufficient number of samples per individual grid. This multifaceted validation framework enables a comprehensive understanding of model behavior under different data partitioning scenarios and temporal scales.

In addition to the regression tasks described above, we conducted an exploratory classification analysis to evaluate the model’s ability to predict categorical pollution levels. For this purpose, the 10 s average PM_2.5_ and PM_1_ concentrations were discretized into five ordinal classes based on their empirical quintiles: Extremely Low PM level, Low PM level, Medium PM level, High PM level, and Extremely High PM level. The data set was randomly split into training (80%) and test (20%) sets using stratified sampling to ensure that the proportion of each class remained similar in both subsets. The LightGBM algorithm was employed. Model performance was assessed using overall accuracy and per-class recall rate on the test set. Overall accuracy measures the proportion of correctly classified samples among all samples. Recall rate quantifies how well the model identifies each class. For a certain class, recall is the number of correctly predicted samples of the class divided by the total number of samples that truly belong to the class. The results were visualized through normalized confusion matrices to illustrate prediction patterns. This classification analysis provides additional insight into the practical utility of audiovisual features for identifying pollution severity levels on urban sidewalks.

## 3 Results

### 3.1 Descriptive Statistics

Table 1 illustrates the descriptive statistics of PM_2.5_ and PM_1_ concentrations, including their temporal variations, providing valuable insights into the distribution and variability of particulate matter at the study site. The mean PM_2.5_ concentration across all time periods was 7.938 μg/m^3^, with a standard deviation (SD) of 2.853 μg/m^3^. For PM_1_ concentrations, the overall mean was 5.269 μg/m^3^ (SD = 2.692 μg/m^3^). Temporal analysis revealed differences between PM concentrations measured in the morning and afternoon. The mean PM_2.5_ and PM_1_ concentration in the morning was 7.186 μg/m^3^ and 4.534 μg/m^3^, respectively, which were lower than the afternoon values of 8.612 μg/m^3^ and 5.928 μg/m^3^, respectively. These trends indicate that both PM_2.5_ and PM_1_ concentrations exhibit temporal variability, with higher air pollution levels typically occurring in the afternoon. Additionally, PM_1_ concentrations were consistently lower than PM_2.5_ concentrations.

**Table 1:** Descriptive Statistics

			percentile				
pollutant	mean	SD	min	25th	50th	75th	max
**PM**_**2.5**_	7.938	2.853	2.369	6.134	7.93	9.722	68.73
**PM**_**2.5**_ **in the morning**	7.186	2.714	2.369	5.555	7.254	9.109	32.677
**PM**_**2.5**_ **in the afternoon**	8.612	2.807	3.15	6.603	8.748	10.242	68.73
**PM**_**1**_	5.269	2.692	0.392	3.388	4.996	6.966	56.533
**PM**_**1**_ **in the morning**	4.534	2.492	0.392	3.014	4.425	6.171	28.418
**PM**_**1**_ **in the afternoon**	5.928	2.693	1.141	3.763	5.804	7.678	56.533

The spatial distribution of PM concentrations was also mapped at the sidewalk segment level, shown in Figure 4. PM_2.5_ and PM_1_ concentrations showed similar spatial patterns, with higher pollution levels concentrated primarily in the south-central part of the study area. Elevated air pollution was observed on sidewalks along the main arterial roads, including Fuzhong First Road, Fuzhong Road, and Fuzhong Third Road. In terms of specific concentration values, the sidewalk segment with the highest PM_2.5_ levels recorded a concentration of 32.28 μg/m^3^, while the lowest value was 3.394 μg/m^3^. The sidewalk segment with the highest PM_1_ levels had a concentration value of 26.062 μg/m^3^, while the lowest value was 1.394 μg/m^3^. After aggregating air pollution values to the sidewalk scale, the gap between the highest and lowest pollution concentrations narrowed.

**Figure 4:**
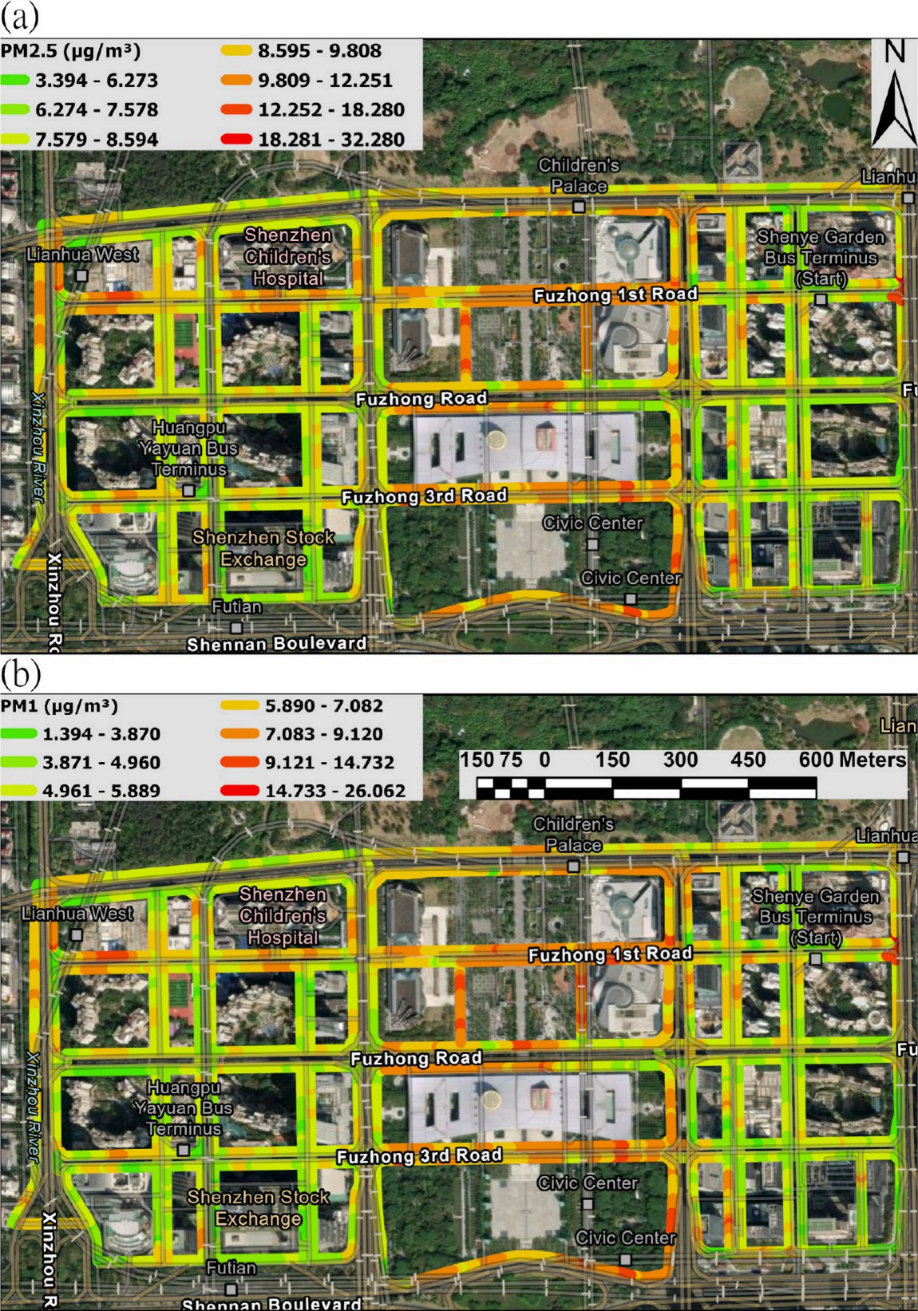
Spatial distribution of concentrations for: (a) PM_2.5_; (b) PM_1_.

### 3.2 Air Pollution Levels Prediction Using the Audiovisual Model

#### 3.2.1 Regression Analysis for Air Pollution Prediction

To evaluate the performance of air pollution prediction using audiovisual variables, we developed an audiovisual model. This model utilized variables extracted from video images, audio recordings, meteorological data, and background air pollution levels while excluding variables derived from geospatial databases. Several machine learning algorithms were employed to compare the predictive accuracy, and the results are summarized in Table 2. Across all validation strategies, temporal aggregations, and pollutant types, the tree-based ensemble models (XGBoost and LightGBM) consistently and substantially outperformed the linear regression. For PM_2.5_ at the 10 s resolution under random CV, LightGBM achieved an R^2^ of 64.13% and an MSE of 2.88, compared to only 23.50% R^2^ for linear regression. Similarly, for PM_1_ under the same conditions, LightGBM attained an R^2^ of 64.68% versus 23.82% for linear regression.This performance gap persisted across all validation strategies, confirming that the complex, nonlinear relationships between audiovisual features and PM concentrations cannot be adequately captured by simple linear models. The performance difference between XGBoost and LightGBM was marginal, with LightGBM showing a slight advantage in most scenarios. Aggregating predictions from 10 s to 1 min consistently and markedly improved model performance across all algorithms and validation strategies. For PM_2.5_ under random CV, LightGBM’s R^2^ increased from 64.13% at 10 s resolution to 80.28% at 1 min resolution, while MSE decreased from 2.88 to 1.32. Similar improvements were observed for PM_1_, with R^2^ increasing from 64.68% to 80.02% under the same conditions. This enhancement can be attributed to the reduction of high-frequency noise inherent in instantaneous measurements and the stabilization of the relationship between the environmental features and pollutant concentrations over longer averaging periods.

**Table 2:** Prediction Performance Using Audiovisual Model

	R^2^			MSE		
pollutant–aggregation units–validation	linear regression	XGBoost	LightGBM	linear regression	XGBoost	LightGBM
**PM_2.5_–10 s–random CV**	23.50%	64.25%	64.13%	6.13	2.87	2.88
**PM_2.5_–10 s–Montecarlo CV**	23.19%	61.98%	63.60%	6.29	3.14	3.01
**PM_2.5_–10 s–spatial route-based CV**	15.22%	39.27%	41.46%	6.88	4.94	4.74
**PM_2.5_–10 s–spatial grid-based CV**	19.10%	55.61%	56.22%	6.09	3.35	3.3
**PM_2.5_–1 min–random CV**	31.80%	78.45%	**80.28%**	4.54	1.44	**1.32**
**PM_2.5_–1 min–Montecarlo CV**	31.89%	79.47%	**81.10%**	4.55	1.37	**1.27**
**PM_2.5_–1 min–spatial route-based CV**	15.86%	43.21%	47.63%	5.62	3.81	3.46
**PM_1_–10 s–random CV**	23.82%	65.14%	64.68%	5.44	2.5	2.53
**PM_1_–10 s–Montecarlo CV**	23.44%	63.36%	64.35%	5.56	2.68	2.61
**PM_1_–10 s–spatial route-based CV**	12.05%	35.27%	36.94%	6.24	4.6	4.46
**PM_1_–10 s–spatial grid-based CV**	19.95%	56.19%	56.55%	5.45	2.99	2.95
**PM_1_–1 min–random CV**	31.43%	78.56%	**80.02%**	4.11	1.29	**1.2**
**PM_1_–1 min–Montecarlo CV**	32.04%	80.20%	**81.58%**	4.06	1.19	**1.1**
**PM_1_–1 min–spatial route-based CV**	13.05%	37.42%	41.25%	5.1	3.7	3.39

The choice of validation strategy had a substantial impact on the estimated model performance, reflecting different aspects of model generalizability. Random CV yielded the most optimiztic estimates, with LightGBM achieving R^2^ values of 64.13% and 64.68% for PM_2.5_ and PM_1_, respectively, at the 10 s resolution. Monte Carlo CV produced comparable results (PM_2.5_: 63.60%, PM_1_: 64.35%), with low variance across the 50 iterations, confirming that the model’s performance is stable across different random data splits and not overly dependent on a particular training set. In contrast, spatial cross-validation provided a more stringent assessment of model generalizability to unseen geographic areas, with notable differences observed between the two spatial partitioning strategies. Under the spatial route-based CV at the 10-s resolution, LightGBM’s R^2^ dropped to 41.46% for PM_2.5_ and 36.94% for PM_1_. This substantial decrease reflects the inherent challenge of macro-level spatial extrapolation, where the model is forced to predict concentrations in entirely new, large-scale routes with potentially different urban morphology and emission sources. However, when employing the finer spatial grid-based CV (100 × 100 m grids), the model’s performance improved significantly, achieving R^2^ values of 56.22% for PM_2.5_ and 56.55% for PM_1_. This improvement suggests that when the spatial partitioning is sufficiently granular and random, the training set captures a more diverse representation of urban microenvironments, allowing the model to generalize much more effectively to unmonitored grids. At the 1 min resolution, spatial CV performance improved further, with LightGBM achieving R^2^ values of 47.63% for PM_2.5_ and 41.25% for PM_1_, suggesting that longer averaging times also enhance spatial generalizability. Both pollutants exhibited similar patterns in response to algorithm choice, temporal aggregation, and validation strategy, indicating that the audiovisual features capture information relevant to different size fractions of fine particulate matter.

To further understand the model’s behavior across different pollution severities, the continuous prediction errors of the LightGBM model were analyzed. The observed 10 s PM concentrations were discretized into five distinct categories—Extremely Low, Low, Medium, High, and Extremely High—based on their empirical quintiles. Figure 5 presents the descriptive statistics of the prediction errors for each bin. The error distributions reveal a clear dependency on the true concentration levels. In the “Extremely Low” and “Low” categories, the mean errors are slightly positive, indicating that the model has a minor tendency to overpredict at the lower end of the spectrum. Additionally, the standard deviations and the ranges between the minimum and maximum errors are relatively small, reflecting highly stable predictions. Conversely, as true concentration levels increase into the “High” and “Extremely High” categories, the mean errors become increasingly negative. The relative scarcity of extreme peak values in the training data causes the model to conservatively underestimate severe pollution spikes.

**Figure 5:**
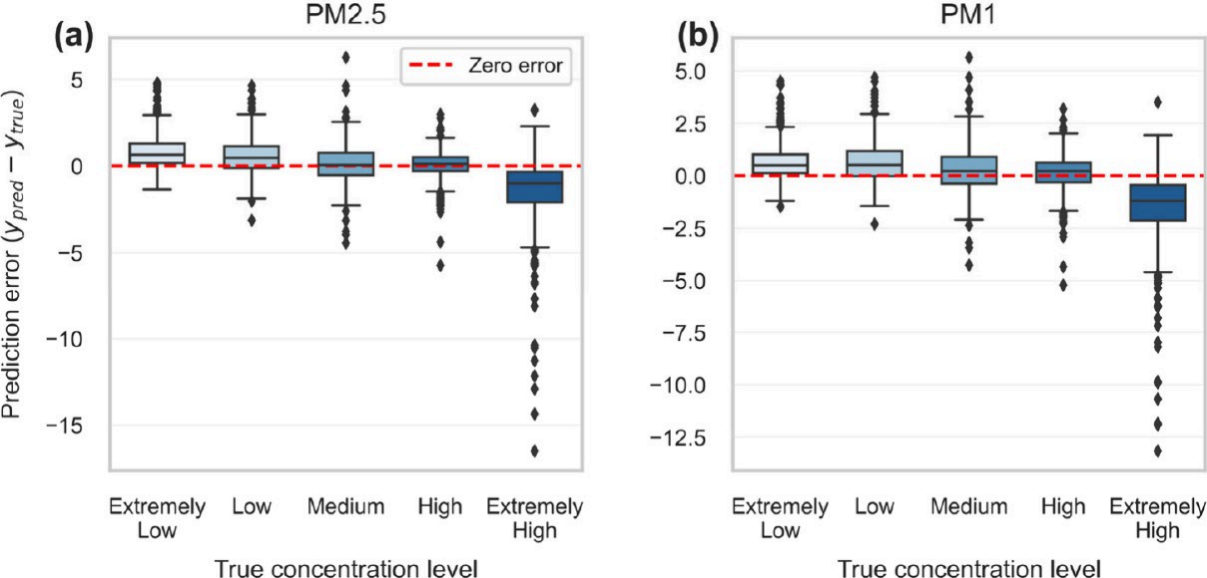
Prediction error for each pollution level of the pollutant: (a) PM_2.5_; (b) PM_1_.

#### 3.2.2 Classification Model for Air Pollution Prediction

While regression models provide continuous concentration estimates, categorical pollution levels are often more intuitive for the general public and more actionable for public health warnings and urban air quality management. As an exploratory extension to the regression analyses, we evaluated the ability of the LightGBM model to classify 10 s PM concentrations into five ordinal classes (Extremely Low, Low, Medium, High, and Extremely High) based on the empirical quintiles of the observed data. The overall classification accuracies were 62.74% for PM_2.5_ and 62.62% for PM_1_. The normalized confusion matrices for both pollutants, which illustrate the prediction performance across different PM levels, are presented in Figure 6. Because the matrices are normalized by true labels (row-wise normalization), the diagonal element in each row directly corresponds to the recall rate for that specific class. For PM_2.5_, the model performed best at the lower end of the spectrum, achieving a recall rate of 78% for the “Extremely Low” category. The model also demonstrated strong capability in identifying elevated pollution risks, with a recall rate of 64% for the “Extremely High” category. The “Medium” category proved the most challenging to classify accurately, yielding a recall rate of 55%. For PM_1_, the classification patterns were highly similar. The model achieved a 79% recall rate for the “Extremely Low” class and a 61% recall rate for the “Extremely High” class. The recall for the “Medium” class was 53%. When the model failed to correctly identify “Extremely High” events, these misclassifications were predominantly limited to the adjacent category (i.e., confusing “Extremely High” with “High”, which occurred 25% of the time for PM_2.5_ and 27% of the time for PM_1_). As expected, the probability of a severe misclassification—such as predicting an “Extremely High” or “High” PM level as an “Extremely Low” or “Low” PM level—is fairly low (e.g., only 5% of true “Extremely High” PM events were predicted as “Low”, and 1% as “Extremely Low”). Overall, the classification results demonstrate that audiovisual data can not only predict continuous PM concentrations but also reliably categorize periods of varying pollution severity on urban sidewalks.

**Figure 6:**
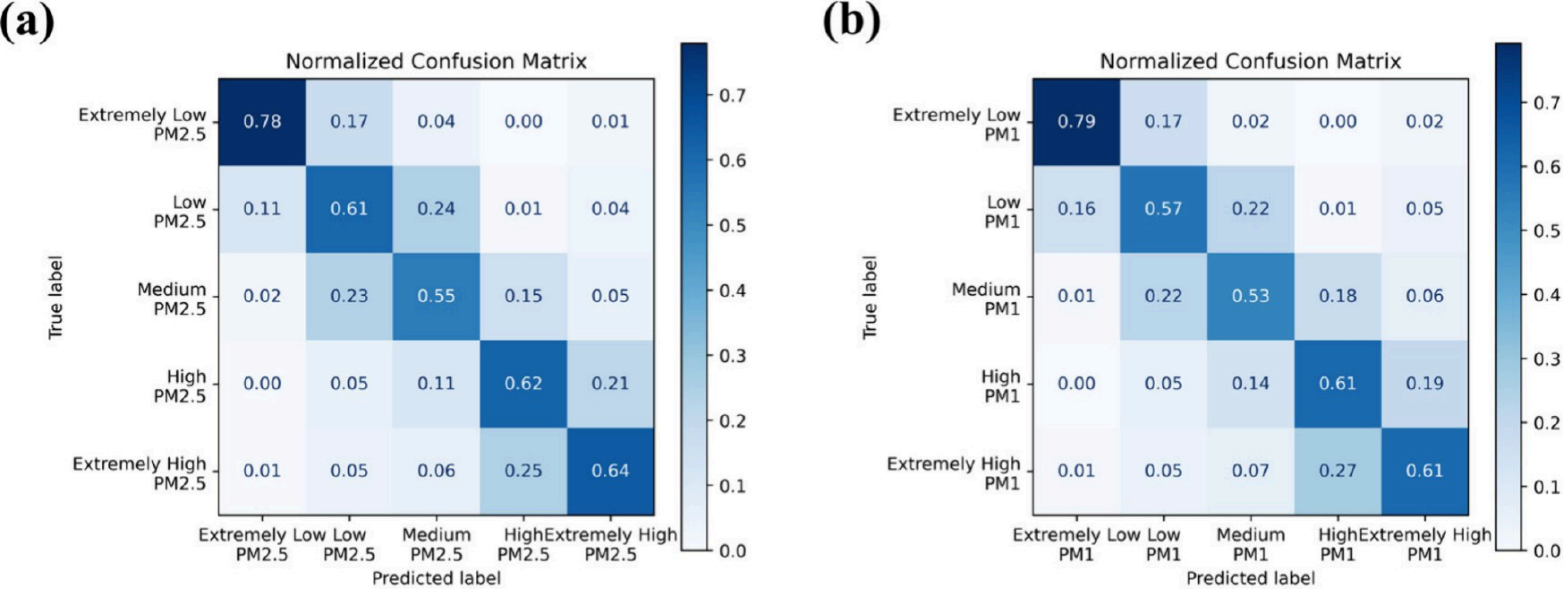
Normalized confusion matrix for the: (a) PM_2.5_; (b) PM_1_ levels classification prediction.

### 3.3 High-Impact Variables Identification

In this study, the SHAP method was utilized to identify the most important features contributing to the prediction of PM concentrations. Figure 7 presents the top 15 variables ranked by global feature importance for air pollution prediction using SHAP values. Each horizontal bar represents a feature, with its length indicating the mean absolute SHAP value, which reflects the overall importance of the feature in driving model predictions. The color gradient along each bar (blue for low values to red for high values) illustrates how the magnitude of the feature value correlates with its impact on the model output—positive SHAP values push predictions higher, while negative values lower them. Features ranked higher have a greater contribution to the model’s predictions. For both PM_2.5_ and PM_1_ prediction, the background PM indicator and meteorological variables emerged as the most significant contributors, consistent with findings from previous studies.^42,43^ Hourly background air pollution level, temperature, and relative humidity ranked first to third, respectively, highlighting their dominant roles in explaining PM variability.

**Figure 7:**
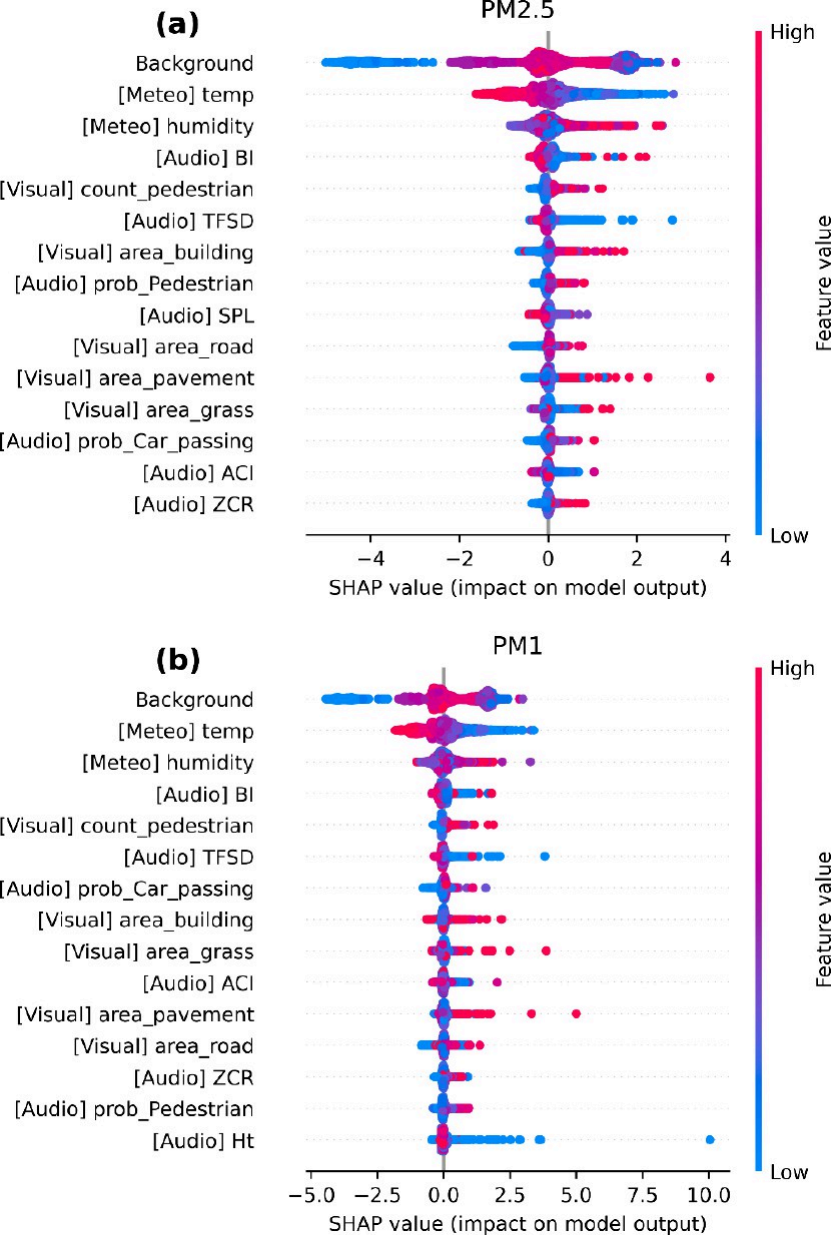
SHAP summary plot of top 15 features in the audiovisual model ranked by global feature importance for: (a) PM_2.5_ concentrations; (b) PM_1_ concentrations in the LightGBM model. The variables on the *y*-axis are ranked from top to bottom based on their mean absolute SHAP values (overall importance). Each dot represents a single data instance. The *x*-axis displays the SHAP value, indicating the impact of that specific feature on the model’s output for that instance (positive values push the prediction higher, negative values push it lower). The color of the dot represents the actual value of the feature for that instance, ranging from low (blue) to high (red). Categorical prefixes (e.g., [Visual], [Audio], [Geo] (geospatial variables), [Meteo] (meteorological variables)) are added to variable names for clarity.

Among the audiovisual predictors, the acoustic indices were consistently important. ***BI*** (bioacoustic index) and ***TFSD*** (time-frequency derivation index) ranked fourth and sixth. They are used to reflect the presence of animal sounds (such as insects and birds)^44,45^ and exhibited negative associations with PM levels in the figure. Higher ***BI*** and ***TFSD*** values indicate greater biophony, suggesting that samples were more likely collected in less anthropogenically influenced environments, such as areas with lower traffic intensity or higher vegetation cover. Other acoustic soundscape indices, such as ***ACI*** (acoustic complexity index) and ***ZCR*** (zero crossing rate), were also identified as important predictor factors. Regarding soundscapes, higher predicted probabilities of car-passing and pedestrian soundscapes were positively associated with PM, consistent with contributions from traffic emissions and resuspension of road dust associated with pedestrian activity.

Visual variables also played an important role in the model predictions. The pedestrian count (***count_pedestrian)*** was among the most influential visual predictors and was positively associated with sidewalk PM concentrations. In addition, a larger road pixel proportion (***area_road***) and pavement pixel proportion (***area_pavement***) were associated with higher predicted PM_2.5_ and PM_1_, likely reflecting an increased proximity to traffic-related emissions and resuspended particles. Furthermore, the building pixel proportion (***area_building***) also showed a notable contribution to PM prediction, which is plausible because the building form affects airflow and thus pollutant dispersion and accumulation.^46^

### 3.4 Combined with Traditional Geographical Variables

To improve predictive performance, we developed a hybrid model by combining audiovisual variables with geospatial variables. Using LightGBM, we trained and evaluated the hybrid model using random cross-validation (10 s aggregation). The R^2^ for PM_2.5_ prediction reached 71.6%, while the R^2^ for PM_1_ prediction reached 70.78%. Compared to the audiovisual model, the R^2^ improved by approximately 7%. This improvement is expected because audiovisual variables primarily capture dynamic, momentary characteristics of the street environment, whereas geospatial variables provide complementary information on persistent spatial context that can influence local PM levels.

The SHAP summary plot of the high-impact features in the hybrid model is shown in Figure 8. In Figure 8(a), the high-impact geospatial variables for PM_2.5_ prediction include ***BuildingVolume_500*** (building volume within 500 m), ***Number_Road_furniture_500*** (number of road furniture POIs within 500 m), ***NDVI_300*** (NDVI within 300 m), ***MeanHeight_400*** (average building height within 400 m), ***Number_Vehicle_service_300*** (number of vehicle service POIs within 300 m), ***NDVI_500***, and ***MeanHeight_300***. In Figure 8(b), the high-impact geospatial variables for PM_1_ prediction include ***BuildingVolume_500***, ***NDVI_500***, ***BuildingVolume_400***, ***Number_Commercial** **&finance_300*** (number of commercial and finance POIs within 300 m), ***Number_Shopping_100*** (number of shopping POIs within 100 m), and ***MeanHeight _300***. Overall, the SHAP results indicate that building morphology, POI-based activity proxies, and greenness contribute meaningfully to explaining urban PM variability.

**Figure 8:**
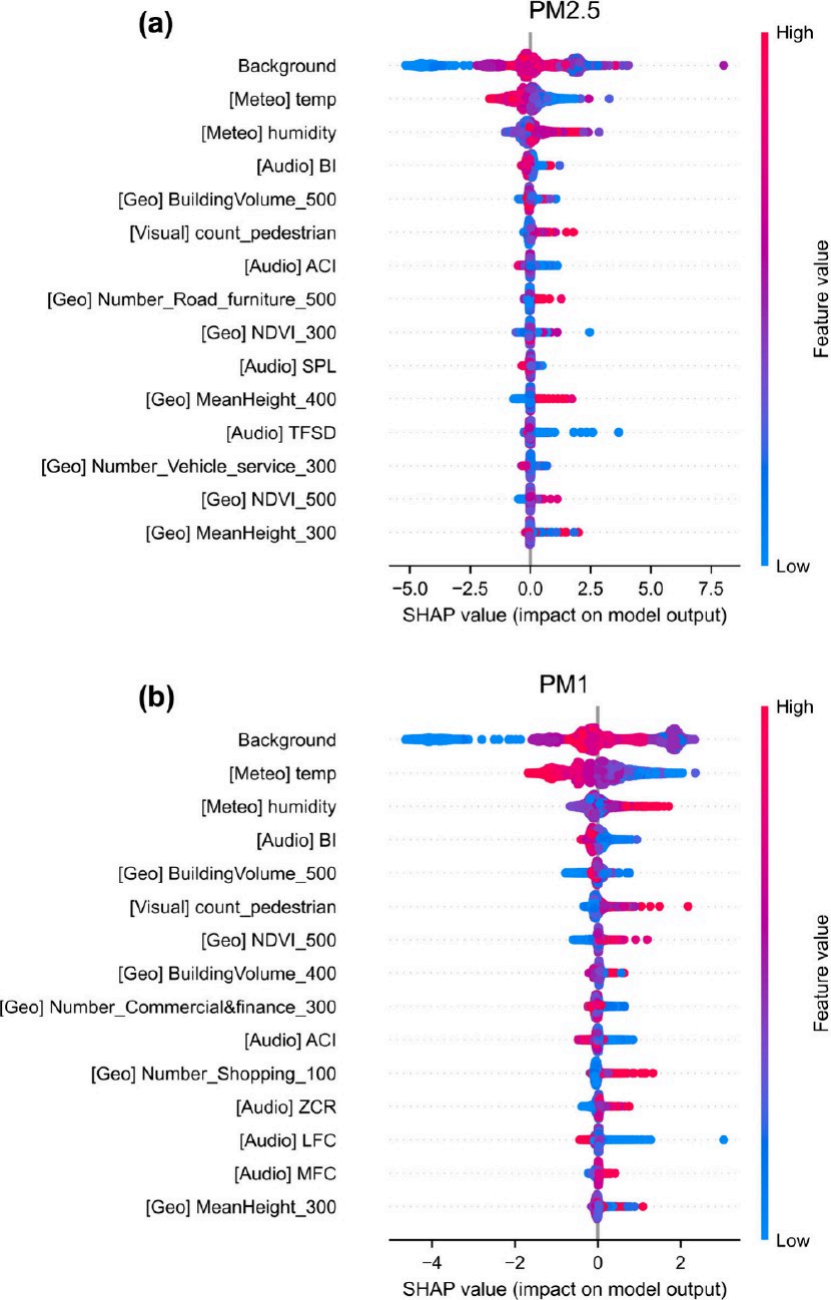
SHAP summary plot of top 15 features in the hybrid model ranked by global feature importance for: (a) PM_2.5_ concentrations; (b) PM_1_ concentrations in the LightGBM model. The visual interpretation of the axes, individual dots, color scheme, and categorical prefixes is identical to that detailed in Figure 7.

The buffer radius associated with the most influential geospatial predictors was primarily 300–500 m. One plausible explanation is that audiovisual variables already capture many near-field environmental conditions, whereas geospatial variables add information about broader neighborhood-scale characteristics that may affect emissions and dispersion. Because audiovisual variables are constrained by the immediate sampling environment, they may be less able to represent the influence of more distant features. Among the top 15 features for PM_2.5_ prediction, more geospatial variables (7) appeared than audiovisual variables (5), whereas for PM_1_ prediction the numbers were equal (6 geospatial vs 6 audiovisual). Notably, however, audiovisual variables remained dominant among the highest-ranked predictors (top 6–7 features) in both models. This finding highlights the unique value of audiovisual variables for PM estimation, as they capture dynamic and highly localized environmental characteristics that static geospatial variables cannot fully represent.

## 4 Discussion

This study developed and evaluated machine-learning models to predict short-term pedestrian-level particulate matter concentrations (PM_2.5_ and PM_1_) on urban sidewalks using features extracted from self-collected street-view videos. The framework integrates dynamic audiovisual features (from images and audio), on-site meteorology (temperature and relative humidity measured by AirBeam3), and a regional background indicator (hourly fixed-site PM_2.5_). Using a mobile monitoring campaign in Shenzhen’s CBD, we demonstrate that readily obtainable sensing modalities can provide meaningful predictive power for near-real-time, fine-scale PM exposure assessment.

For air pollution prediction, across algorithms, tree-based gradient boosting models (LightGBM and XGBoost) consistently outperformed linear regression, indicating that the relationships between PM levels and audiovisual descriptors are nonlinear and characterized by interactions. The specific characteristics of our data led us to prioritize these advanced models over other approaches. First, our data set is highly heterogeneous, comprising categorical probabilities, discrete counts, and continuous physical measurements. Tree-based models are highly robust in accommodating such diverse tabular data. Second, these models integrate seamlessly with AutoML frameworks like FLAML (used in this study), allowing for highly efficient hyperparameter optimization. Finally, by coupling LightGBM/XGBoost with SHAP, we achieved an optimal balance between high predictive accuracy and the granular interpretability required to disentangle the individual contributions of novel audiovisual features, an advantage that “black-box” deep learning models lack. Regarding specific performance, for the audiovisual model (excluding geospatial variables), tree-based algorithms achieved strong predictive performance under random cross-validation and comparable performance under Monte Carlo resampling, suggesting that results were not overly sensitive to a particular random split. As expected, performance declined under spatial cross-validation, which provides a more conservative and realistic estimate of generalization to unseen locations. The remaining predictive power under spatial blocking indicates that the extracted audiovisual and meteorological cues capture transferable signals, but also highlights the difficulty of extrapolating sidewalk-level PM models across heterogeneous microenvironments with different emission intensities, street configurations, and dispersion conditions. Temporal aggregation had a pronounced effect on model performance. Aggregating 10 s observations to 1 min averages improved R^2^ and reduced MSE for both pollutants across models and validation strategies. As further detailed in our sensitivity analysis (Supporting Information Section 5), model performance peaked at a 3 min aggregation window.

A key contribution of this work is the explicit incorporation of audio information and the finding that audio-derived variables were repeatedly among the most influential predictors alongside background pollution and meteorology. This suggests that urban sound carries useful information about traffic dynamics and human activity intensity that is not always captured visually, especially in complex street settings, where occlusion, lighting, and camera perspective can limit object detection quality. SHAP analyses indicate that audio variables contribute substantially to predictions. Indices such as ***BI*** and ***TFSD*** showed negative relationships with PM levels. In contrast, higher predicted probabilities of car passing and pedestrian soundscapes were associated with higher PM, consistent with traffic emissions and resuspension processes in active sidewalk environments. Visual variables also contributed meaningfully to prediction. Features such as pedestrian counts and road/pavement pixel proportions were positively associated with predicted PM, consistent with these variables acting as proxies for street activity level, proximity to vehicle lanes, and microenvironmental conditions that promote emissions and resuspension. Building-related visual features also appeared among important predictors, which is plausible because the surrounding built form affects near-road ventilation and pollutant accumulation. Combining audiovisual features with buffer-based geospatial variables improved performance, confirming that the static spatial context provides complementary information that audiovisual clips cannot fully encode.

Monitoring urban air pollution is critical to safeguarding public health. It provides essential data to inform public policies and urban planning that aim to reduce pollution levels. However, fixed monitoring stations, typically built and maintained by governments, are costly to install and operate. As a result, these stations are often limited to specific locations and cannot provide air pollution monitoring with a fine spatiotemporal resolution. Deploying networks of low-cost sensors can complement fixed stations and offer more granular data, but the purchase and calibration costs of these sensors remain prohibitively high. The audiovisual model proposed in this study for air pollution prediction offers a promising solution to these challenges. The predictor variables in the audiovisual model are primarily derived from videos, which can be recorded by using cameras or even smartphones. These devices are far more accessible and affordable than traditional air pollution monitoring devices including low-cost sensors. In terms of practical application, one potential direction is to significantly enhance the spatiotemporal resolution of urban air pollution monitoring and prediction by leveraging both crowdsourced audiovisual data and existing urban traffic camera networks. Specifically, videos voluntarily recorded and uploaded by citizens via smartphones can be combined with video data from an extensive network of fixed traffic cameras deployed on roads. These heterogeneous sources of video data can be processed into predictive models that enable high-resolution, real-time air pollution monitoring, dramatically expanding the coverage of traditional monitoring systems.

Several limitations must be acknowledged. First, due to experimental cost constraints, the monitoring campaign was conducted only in the central business district (CBD) of Shenzhen, China, and was not extended to other areas of Shenzhen or other cities. This limitation arises partly because the study focused on air pollution on sidewalks, which is difficult to monitor over large areas by using pedestrian-carried instrumentation. In contrast, previous studies often utilized vehicle-mounted devices for mobile air pollution monitoring of roads. To validate the generalizability of the proposed model, future research should expand the geographic scope and duration of data collection to confirm the broader applicability of the methodology. Second, the audiovisual data in this study were collected using panoramic cameras. The performance of the prediction models may be influenced by the quality of the video images and captured sound. Future research could incorporate specialized sound acquisition equipment to improve the data quality. Additionally, advancements in techniques for extracting traffic dynamics features, such as vehicle speed estimation,^47^ could be integrated into the framework to further enhance model performance.

In conclusion, we proposed a novel framework for predicting short-term air pollution levels on urban sidewalks using audiovisual variables extracted from self-collected videos. Advanced machine-learning models were employed for both prediction and variable interpretation. This study demonstrates the potential to complement traditional fixed-site monitoring systems and supports urban policymakers in making timely, data-driven decisions for air-quality management and public health protection.

## Supplementary Material



## Notes

Air pollution monitoring device calibration, soundscape deep learning process and statistical description, details about predictor variables, feature selection in the hybrid model, and sensitivity analysis of temporal resolution (PDF)
