# Supplementary material for: Integrating Audiovisual Data for Short-Term Particulate Matter Concentration Prediction on Urban Sidewalks
Source: Environ Sci Technol. 2026 Jul 22;60(30):21011–23. doi: 10.1021/acs.est.6c03195 (PMC13449881; doi:10.1021/acs.est.6c03195)
Supplement: Supplementary file 1 [file es6c03195_si_001.pdf]

# Supporting Information

Title: Integrating Audiovisual Data for Short-term Particulate Matter Concentration Prediction on Urban Sidewalks

*Xujing Yu[1], Jun Ma[1]*, Waishan Qiu[1], Wei He[2], Feifeng Jiang[3]*

Corresponding author. E-mail address: junma@hku.hk (J. Ma).

1. Department of Urban Planning and Design, Faculty of Architecture, The University of Hong Kong, Hong Kong, China
2. State Key Laboratory of Information Engineering in Surveying, Mapping and Remote Sensing, Wuhan University, Wuhan, China
3. Department of Geography, Hong Kong Baptist University, Hong Kong, China

Summary: 15 pages, 8 figures and 3 tables

Contents of Supporting Information file:

Section 1: Air Pollution Monitoring Device Calibration
Section 2: Soundscape Deep Learning Process
Section 3: Details About Predictor Variables
Section 4: Feature Selection in the Hybrid Model
Section 5: Sensitivity Analysis of Temporal Resolution

## 1. Air Pollution Monitoring Device Calibration

The air quality monitoring device used in this study was the AirBeam3 (https://www.habitatmap.org/airbeam, HabitatMap, United States of America). Four AirBeam3 devices were utilized during the monitoring campaign. According to official laboratory tests (https://www.habitatmap.org/airbeam/users-guide#performance), the AirBeam3 performs well in measuring $PM_1$ and $PM_{2.5}$ concentrations. To further validate the reliability of the AirBeam3 devices, we conducted additional tests and calibrations.

Firstly, we evaluated the performance of the four AirBeam3 devices (labeled A through D) by comparing their PM measurements with those of a regulatory-grade instrument, the Profiler Model 212 (MetOne Instruments, Inc.). The Profiler Model 212 is a highly accurate optical particle counter widely used in air pollution monitoring [1]. Over 30 hours of measurement, the $PM_{2.5}$ and $PM_1$ concentrations recorded by all four AirBeam3 devices exhibited trends consistent with those of the standard instrument, as shown in **Figure S 1**. This consistency demonstrated the reliability of the AirBeam3 devices for measuring $PM_{2.5}$ and $PM_1$ concentrations.

(a)

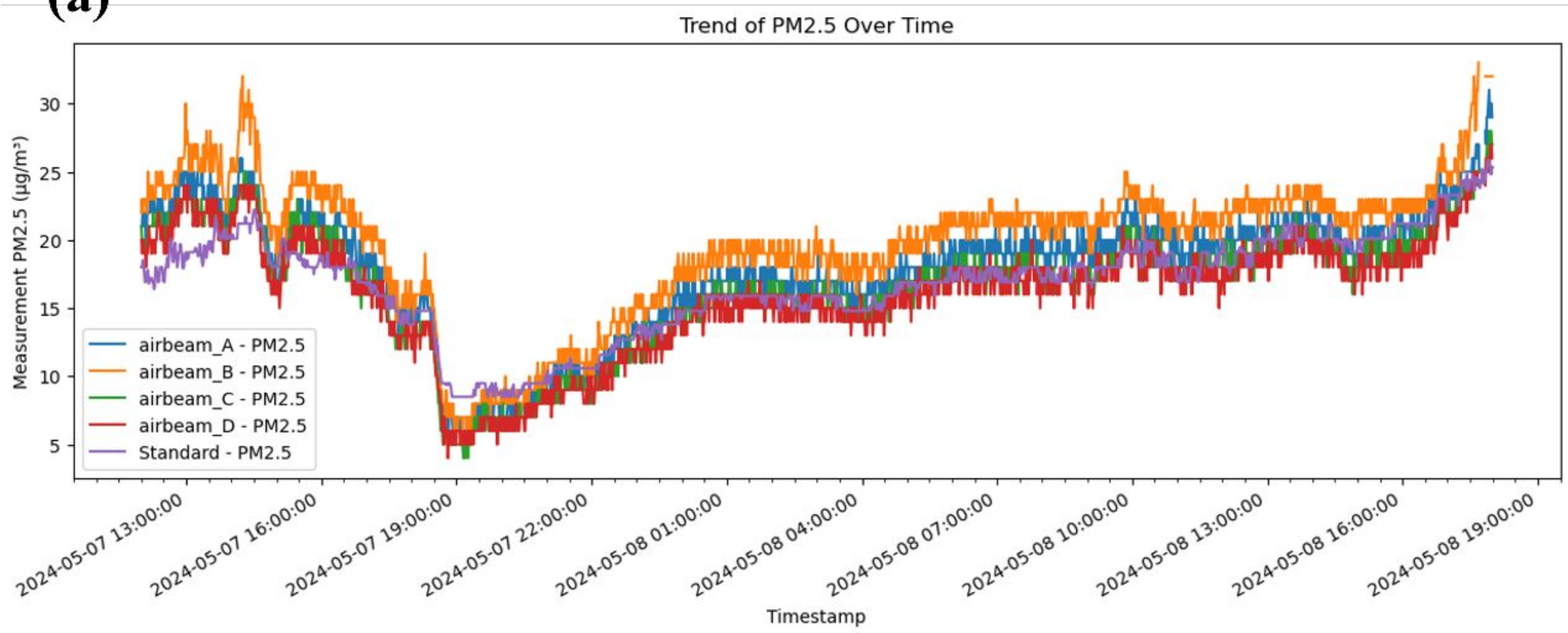


(b)

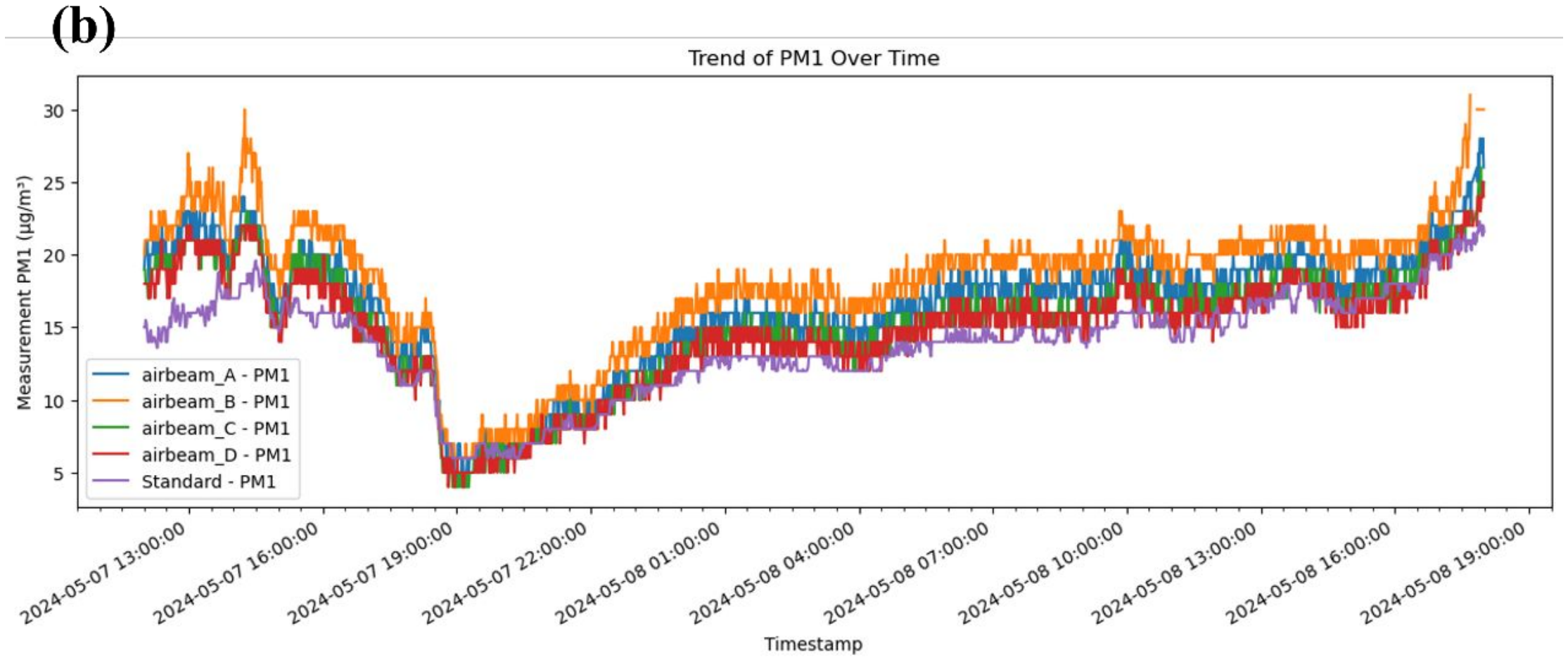


Figure S 1 Comparison of: (a) $PM_{2.5}$; (b) $PM_1$ concentrations measured by the Airbeam3 instruments with those measured by the standard instrument during instrument testing

To improve the accuracy of the AirBeam3 measurements, we developed eight linear regression formulas (four devices and two pollutant types), as shown in **Figure S 2** and **Figure S 3**. In these calibration equations, the X-axis represents the PM concentration values measured by the AirBeam3 device, while the Y-axis represents the PM concentration values measured by the standard instrument. The fitting $R^2$ coefficients for all comparisons between the AirBeam3 devices and the Profiler Model 212 exceeded 90%, indicating a strong linear relationship and reliable calibration performance. The AirBeam3 utilizes a Plantower PMS7003 sensor. According to the South Coast Air Quality Management District (SCAQMD) AQ-SPEC program (https://www.habitatmap.org/blog/evaluation-proves-airbeam3-most-accurate-airbeam-ever), which evaluated 75 instruments, the AirBeam3 was ranked among the most accurate considering both field and laboratory assessments. This support that AirBeam3 provides a stable relative response to particulate matter.

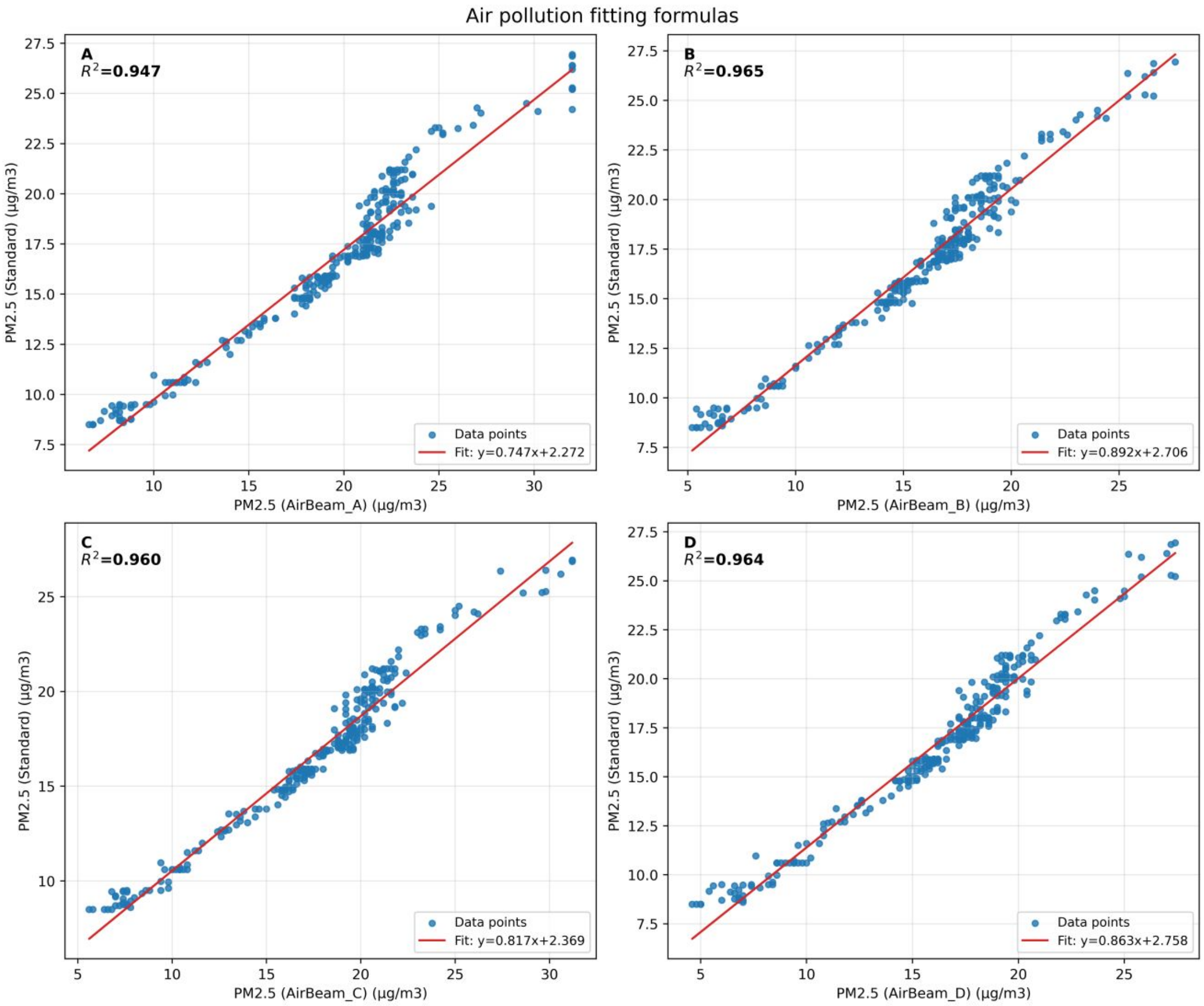


Figure S 2 Fitting formulars for $PM_{2.5}$ concentrations

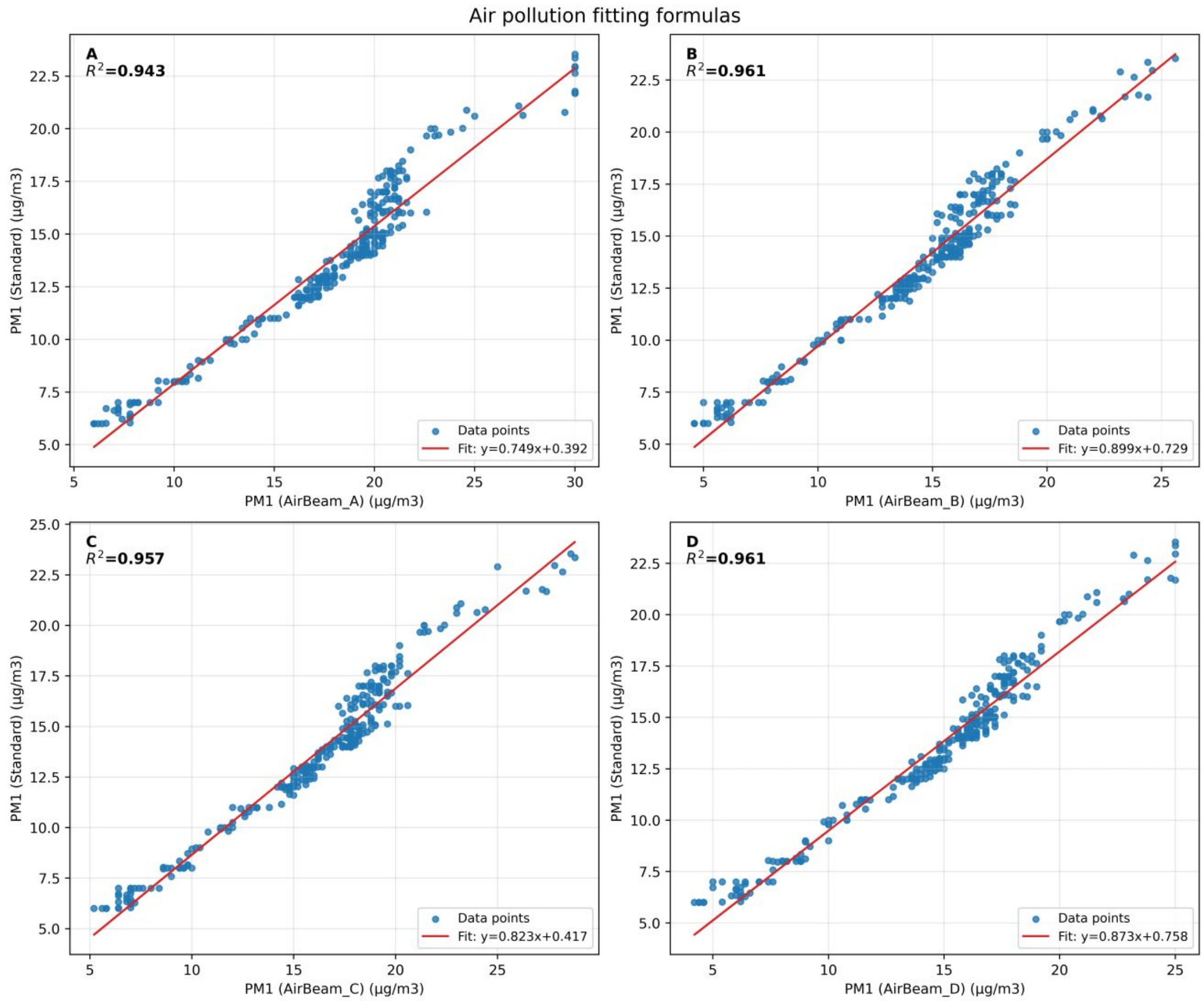


Figure S 3 Fitting formulars for $PM_1$ concentrations

The pollutant data points collected during the monitoring campaign were calibrated using the corresponding formulas derived from the regression analysis, ensuring improved accuracy in the reported PM measurements.

## 2. Soundscape Deep Learning Process and Statistical Description

Some general acoustic indices can be calculated using existing Python toolkits. However, there are no specific indicators or tools available for analyzing the soundscape of urban traffic or sidewalks. To reflect the diversity of soundscape types on urban sidewalks, we performed a deep learning-based process for soundscape prediction. This process was informed by the studies of Verma et al. [2,3], and the framework is illustrated in **Figure S 4**. Firstly, we selected and downloaded sound clips that represented the soundscapes of urban sidewalks from **AudioSet** [4], a well-known sound dataset. AudioSet contains 632 audio event classes that are manually annotated in the form of 10-second clips sourced from YouTube videos. It provides a comprehensive collection of human and animal sounds, as

well as other common environmental sounds. The definitions of these sound classes are available in the AudioSet ontology (https://research.google.com/audioset/ontology/index.html). For this study, we selected a total of 15 classes, encompassing 2,103 audio samples. These samples were reclassified into 7 soundscape types relevant to our research objectives:

1. **Ambient**: Sounds from background noise, such as wind.
2. **Animals**: Sounds from urban wildlife, such as birds and insects, which are typically linked to vegetation and a natural environment.
3. **Car horn**: Reflecting honking sounds from vehicles, indicative of traffic congestion.
4. **Car engine**: Sounds arising from vehicular engines, representing urban traffic.
5. **Car passing**: Noise generated by moving vehicles, which contributes to the traffic soundscape.
6. **Construction**: Urban construction noise, which may also correlate with pollutant emissions from construction activities.
7. **Pedestrian**: Noise from human activity on sidewalks, which can increase exposure to dust and pollution.

Next, we utilized a pretrained deep learning model and fine-tuned it for our urban sidewalk soundscape classification task. Finally, the trained model was employed to process the audio clips collected from the study area, predicting the probability of the existence of the 7 predefined soundscape types.

| Aspect | Soundscape type for the study | Classes from the Audioset |
|---|---|---|
| Environment | Ambient | Wind; Silence |
| | Animals | Bird vocalization, bird call, bird song; Insect |
| Machine | Car horn | Vehicle horn, car horn, honking; Air horn, truck horn |
| | Car engine | Engine starting; Medium engine (mid frequency) |
| | Car passing | Car passing by; Motor vehicle (road) |
| | Construction | Hammer; Jackhammer; Power tool |
| Human generated | Pedestrian | Hubbub, speech noise, speech babble; Children playing |

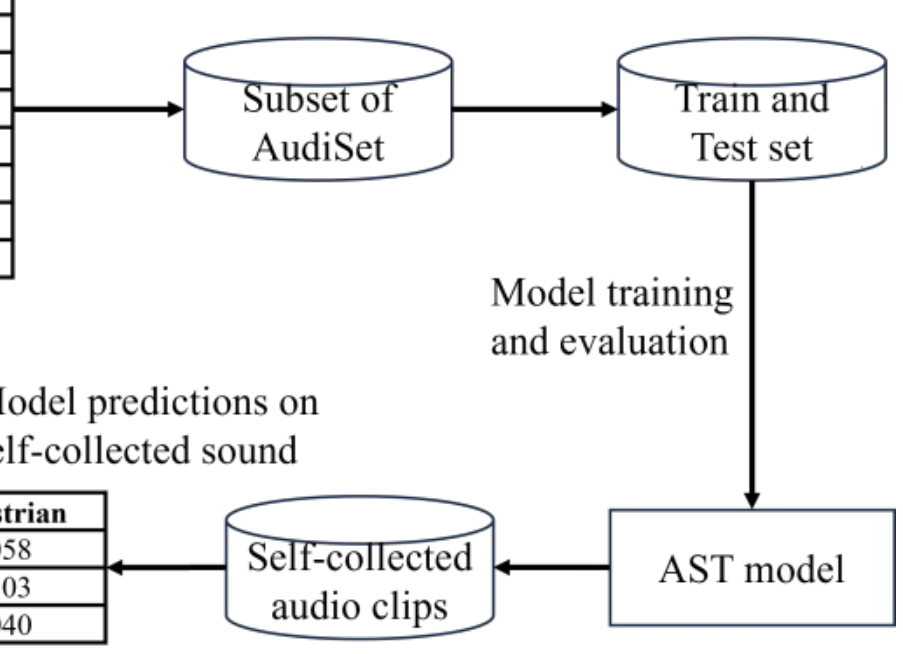


| Audio samples | Ambient | Animals | Car_horn | Car_engine | Car_passing | Construction | Pedestrian |
|---|---|---|---|---|---|---|---|
| Sample#1 | 0.177 | 0.234 | **0.293** | 0.105 | 0.095 | 0.039 | 0.058 |
| Sample#2 | **0.353** | 0.300 | 0.041 | 0.041 | 0.121 | 0.041 | 0.103 |
| Sample#3 | 0.215 | **0.509** | 0.035 | 0.045 | 0.116 | 0.039 | 0.040 |

Figure S 4 The framework demonstrating deep learning process for urban sidewalk soundscape predictions.

The model used for fine-tuning in this study is **Audio Spectrogram Transformer (AST)** model. The AST model applies a Vision Transformer to audio data by converting audio into spectrogram images, achieving state-of-the-art results for audio classification [5]. For model training, 70% of the data was used for training, while the remaining 30% was reserved for validation (test size = 0.3). The number of epochs was set to 50, indicating the number of complete passes the model made over the training dataset. The learning rate was configured to 5.00E-05. To assess the performance of the model, several evaluation metrics were employed, including categorical cross-entropy loss, accuracy, recall, precision, and

F1-score. The equations for these metrics are shown in **Equations** (1)-(5):

$$Loss = -\sum_{j=1}^{n} y_j \log\left(\hat{y}_j\right) (1)$$

$$\text{Accuracy} = \frac{\sum_{j=1}^{n} \text{TP}_j}{\text{T}} (2)$$

$$\text{Precision} = \frac{1}{\sum_{j=1}^{n} w_j} \sum_{j=1}^{n} \left( w_j \frac{\text{TP}_j}{\text{TP}_j + \text{FP}_j} \right) (3)$$

$$\text{Recall} = \frac{1}{\sum_{j=1}^{n} w_j} \sum_{j=1}^{n} \left( w_j \frac{\text{TP}_j}{\text{TP}_j + \text{FN}_j} \right) (4)$$

$$\text{F1 Score} = 2 \times \frac{\text{Precision} \times \text{Recall}}{\text{Precision} + \text{Recall}} (5)$$

Categorical Cross-Entropy Loss measures the difference between the predicted soundscape class probabilities $\hat{y}_j$ and the ground-truth probabilities $y_j$ for each class. n represents the total number of classes. $TP_j$ is the number of samples correctly classified in soundscape class $j$, $T$ is the total number of samples. $FP_j$ represents the number of samples erroneously predicted in soundscape class $j$, $FN_j$ is the number of samples belonging to soundscape class $j$ but incorrectly classified into other classes, and $w_j$ is the weight assigned to soundscape class $j$, determined by the number of true samples for each class. In general, a lower Loss and higher Accuracy, Precision, Recall, and F1-score indicate better model performance.

The model evaluation results are presented in **Figure S 5**. As training progresses, the validation loss decreases and eventually stabilizes at a relatively low level. Meanwhile, the accuracy and other evaluation metric curves exhibit a trend opposite to the loss curves. Initially, there is a significant increase in these metrics, indicating that the model is effectively learning to make more accurate predictions. As training continues, the curves generally stabilize at a certain level, reflecting the model's convergence. The final model achieved an accuracy and other evaluation scores near 90%, demonstrating strong performance in soundscape prediction. Consequently, the best-performing model from the training epochs was selected to predict the existence of the 7 soundscape types in the study area and calculate their respective probabilities.

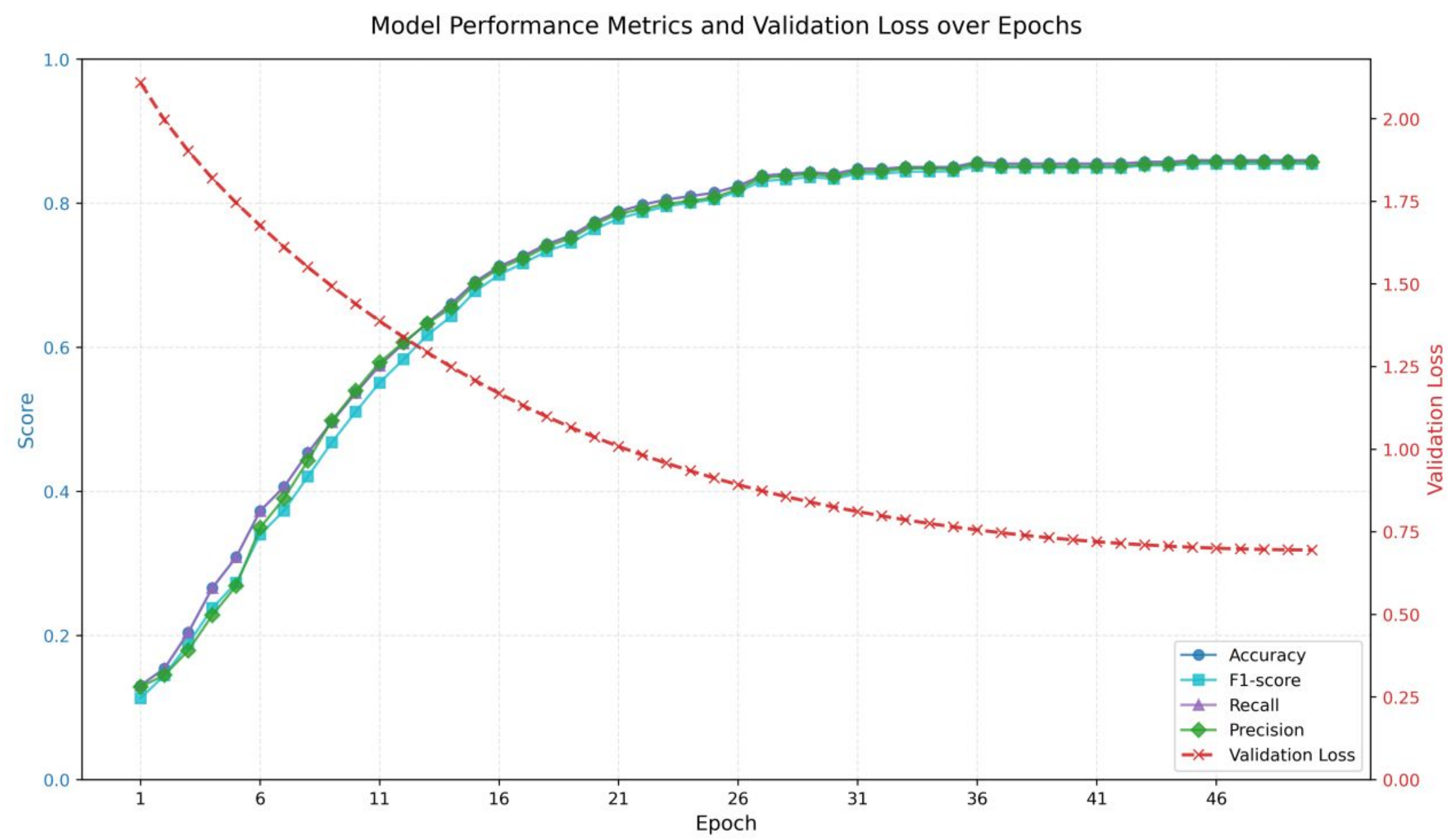


Figure S 5 Model evaluation results

To provide a comprehensive overview of the acoustic environment captured during the monitoring campaign, we present descriptive statistics for the probabilities of the seven soundscape types predicted by the fine-tuned AST model across 10-second audio clips, shown in TableS 1. The results indicate that ***Ambient*** sounds (e.g., wind, background environmental noise) have the highest mean probability (0.40), suggesting that general background sounds constitute the predominant acoustic component throughout most of the monitoring period. Among the remaining soundscape categories, ***Animals*** (e.g., birds, insects) and ***Car_passing*** exhibit the highest mean probabilities, at 0.20 and 0.15 respectively. The relatively high probability of animal sounds was somewhat unexpected given the urban context of the study area. However, this finding can be attributed to two factors. First, the monitoring campaign was conducted in July, which corresponds to the summer season when insect and bird activity is at its peak in subtropical cities like Shenzhen. Second, many of the monitoring routes, particularly those adjacent to parks and along tree-lined streets, feature dense roadside vegetation. In sections with lower traffic volume, the sounds of cicadas and birds became distinctly prominent in the audio recordings, contributing to the elevated animal sound probabilities. Other soundscape categories show substantially lower mean probabilities, indicating that these sources are more localized within the study area. Notably, the magnitude of a soundscape category's mean probability does not necessarily correspond to its importance in predicting air pollution concentrations. The wide ranges and standard deviations observed for several categories demonstrate that the soundscape is highly variable across the study area, capturing a broad spectrum of urban acoustic environments.

TableS 1 Descriptive statistics of predicted soundscape probabilities

| Soundscape | Mean | Std | Min | 25% | 50% | 75% | Max |
|---|---|---|---|---|---|---|---|
| prob_Ambient | 0.40 | 0.19 | 0.01 | 0.25 | 0.40 | 0.55 | 0.90 |
| prob_Animals | 0.20 | 0.17 | 0.01 | 0.08 | 0.15 | 0.28 | 0.92 |
| prob_Car_horn | 0.08 | 0.08 | 0.00 | 0.04 | 0.06 | 0.09 | 0.78 |
| prob_Car_passing | 0.15 | 0.08 | 0.01 | 0.09 | 0.13 | 0.19 | 0.64 |
| prob_Pedestrian | 0.05 | 0.04 | 0.01 | 0.03 | 0.04 | 0.06 | 0.62 |
| prob_Car_engine | 0.06 | 0.03 | 0.01 | 0.04 | 0.06 | 0.08 | 0.36 |
| prob_Construction | 0.05 | 0.03 | 0.00 | 0.03 | 0.04 | 0.06 | 0.40 |

## 3. Details About Predictor Variables

The details of the predictor variables used in the audiovisual model are presented in **TableS 2**. In total, the model includes 36 predictor variables. To assess potential multicollinearity among these variables, correlation heat maps were generated, shown in **Figure S 6**. The analysis revealed that the absolute values of the correlation coefficients between all variables, except the meteorological variable, do not exceed 0.75. This indicates only weak multicollinearity among the variables. Furthermore, the tree-based machine learning algorithms employed in this study are capable of effectively addressing this weak multicollinearity.

TableS 2 Description of predictor variables in the audiovisual model

| Aspects | | Variables | Number | Extraction methods |
|---|---|---|---|---|
| Visual | Segmented area | Sky, building, tree, grass, road, pavement, car, bus, truck | 9 | Use panoptic segmentation techniques for image segmentation and object detection |
| | Detected object number | Person, car, bicycle, bus, truck | 5 | |
| Audio | Sound pressure level | Sound pressure level (SPL) | 1 | Use scikit-maad Python package for calculation |
| | Alpha acoustic indices | The cover of the spectrogram above a threshold for three bandwidths: low frequency band (LFC), medium frequency band (MFC) and high frequency band (HFC) | 3 | |
| | | Bioacoustics index (BI), acoustic complexity index (ACI), time frequency derivation index (TFSD) | 3 | |
| | | The total number/cover of the regions of interest (ROItotal/ROIcover) | 2 | |
| | | Entropy of spectral maxima (EPS), temporal entropy of the audio (Ht) | 2 | |
| | Temporal acoustic features | Zero crossing rate feature of an audio signal (ZCR) | 1 | |
| | Predicted soundscape types | Probability of audio clip being classified as sounds of vehicle horn, vehicle passing, vehicle engine, construction, pedestrians, animals, and the ambient | 7 | Use pretrained deep learning model (Audio Spectrogram Transformer) for fine-tune and inference |
| Background air pollution | | Hourly fixed-site $PM_{2.5}$ concentrations | 1 | Air pollution monitoring station |
| Meteorology | | Relative humidity, temperature | 2 | Collect with AirBeam instruments |

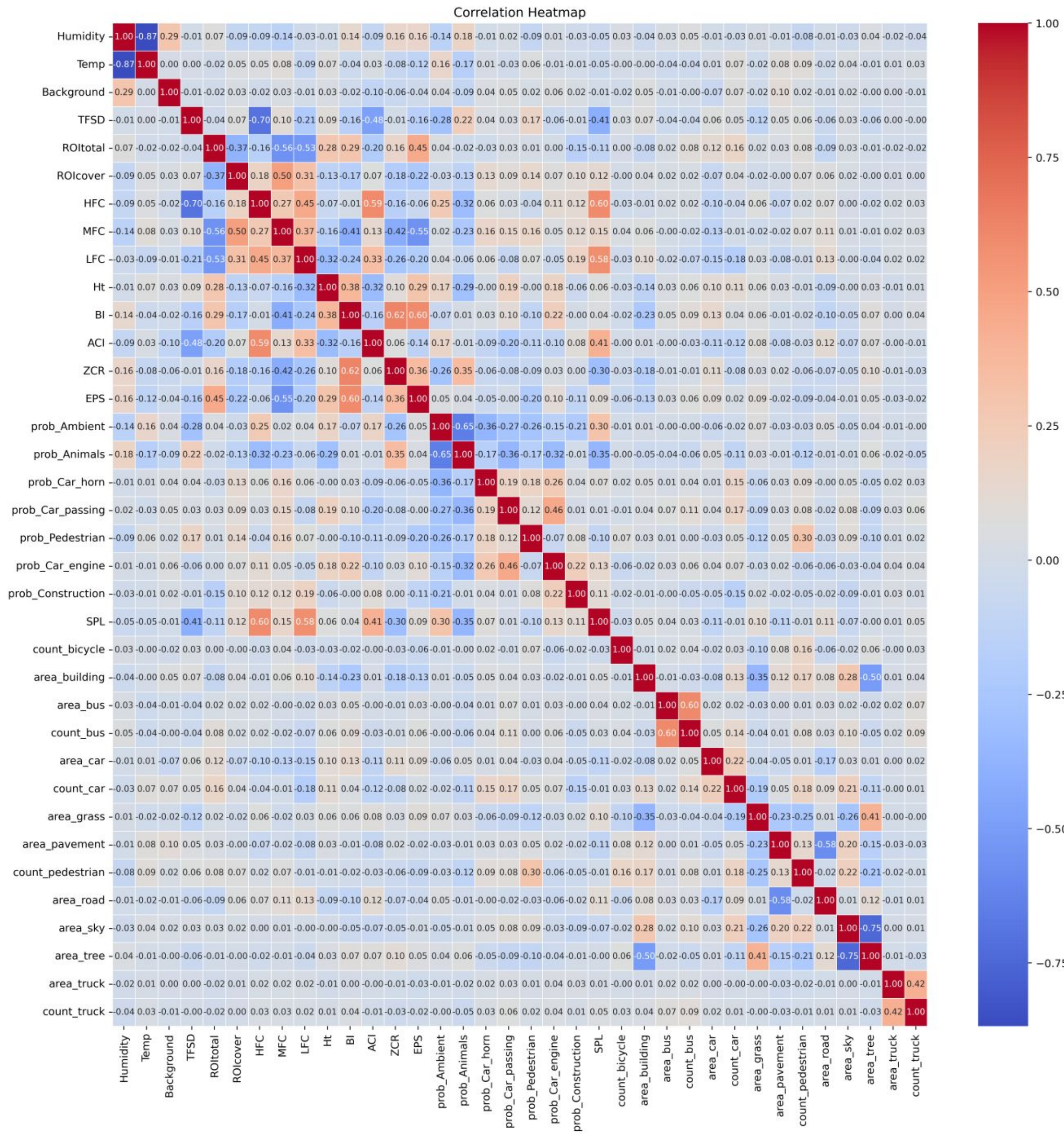


Figure S 6 Correlation among predictor variables

In the hybrid model, discussed in Section 2.3 **("General predictor variables")**, additional geospatial variables were incorporated into the audiovisual model, forming the hybrid model. The geospatial variables included building morphology, points of interest (POIs), and land cover data. These variables were selected based on findings from previous studies [6–8]. To capture the effects of geospatial variables at finer spatial scales, buffer-based feature augmentation was applied. Buffer radii ranged from 100 meters to 500 meters (in increments of 100 meters). In total, 45 geospatial variables were incorporated alongside the audiovisual variables in the hybrid model.

TableS 3 Description of geospatial variables

| Aspect | Variables | Sources | Buffer | Number |
|---|---|---|---|---|
| Building | Mean of building height | AMAP, government open building height data | 100, 200, 300, 400, 500 m | 5 |
| | Standard deviation of building height | | | 5 |
| | Building volume | | | 5 |
| POIs | Count of shopping POI | AMAP | | 5 |
| | Count of road furniture POI (e.g. traffic light) | | | 5 |
| | Count of vehicle service POI | | | 5 |
| | Count of catering POI | | | 5 |
| | Count of commercial & finance POI | | | 5 |
| Land cover | NDVI | Sentinel-2A satellite image | | 5 |

## 4. Feature Selection in the Hybrid Model

The hybrid model incorporated a large set of geospatial variables in addition to the audiovisual features. To identify the most informative subset of variables for predicting $PM_{2.5}$ and $PM_1$ concentrations, we employed Recursive Feature Elimination with Cross-Validation (RFECV) as implemented in the Python *scikit-learn* package. Recursive Feature Elimination (RFE) is a greedy optimization algorithm that aims to select a subset of features by recursively considering smaller and smaller sets. Through RFE, the estimator is trained on the initial set of features, and the importance of each feature is obtained. The least important feature(s) are pruned from the current set of features and the procedure is recursively repeated on the pruned set until the desired number of features to select is reached. We applied RFECV separately for the two target variables ($PM_{2.5}$ and $PM_1$) using the following configuration:

- Estimator: LightGBM regressor. The hyperparameters of the LightGBM model were optimized using the FLAML (Fast and Lightweight AutoML) library [9]. FLAML performs an automated hyperparameter search to find the best configuration for the given dataset and task.
- Cross-validation: 5-fold stratified cross-validation (CV) was used within the RFECV loop. The data was split into five folds, and for each candidate feature subset, the model was trained on four folds and evaluated on the held-out fold.
- Scoring metric: $R^2$.

The RFECV process was implemented using the *RFECV* class from *sklearn.feature_selection*. To ensure computational efficiency, we leveraged the built-in support for feature importance from the LightGBM estimator. **Figure S 7** shows the process of feature selection by the RFE. There is a total of 81 candidate predictors**.** The

RFECV analysis yielded different optimal feature set sizes for the two pollutants. For $PM_{2.5}$, the optimal number of features was 69, while for $PM_1$, the optimal number of features was 41. After determining the optimal feature sets, the corresponding subsets of variables were used for all subsequent analyses reported in the main manuscript, including the final hybrid model training and the SHAP-based interpretation of feature importance.

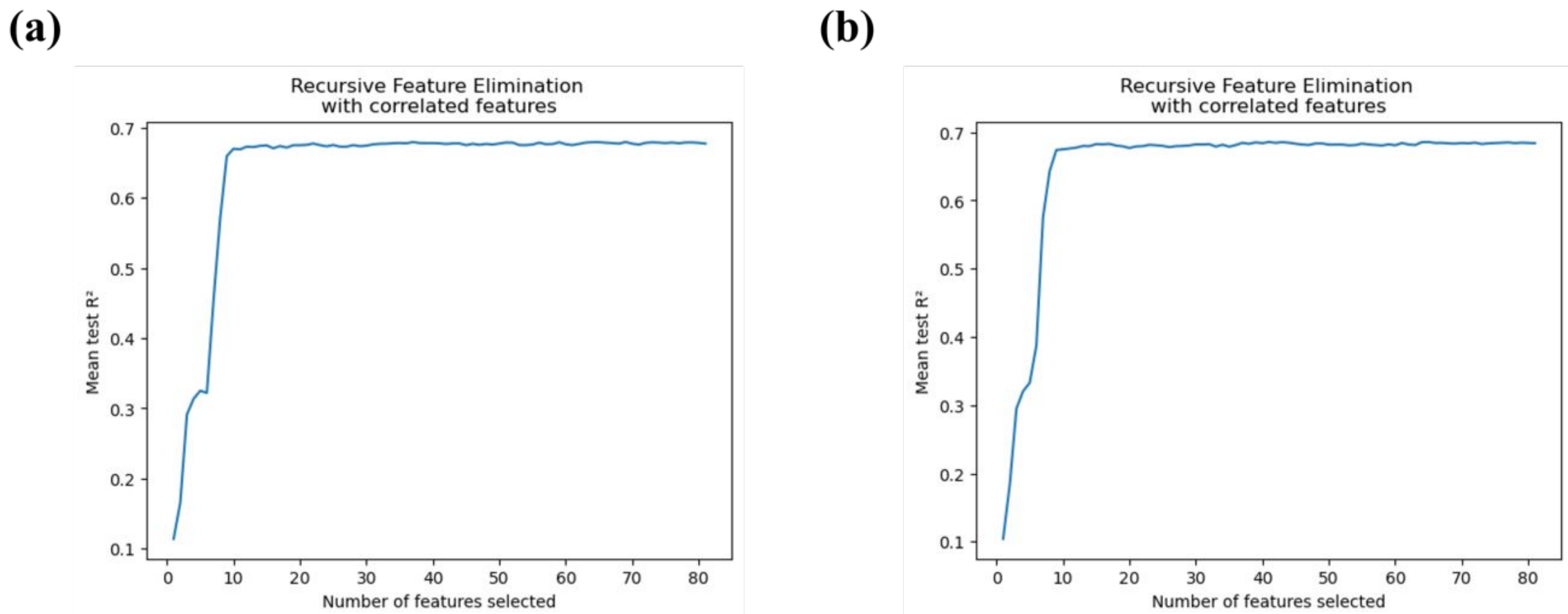


Figure S 7 The process of feature selection by the RFE for: (a) $PM_{2.5}$; (b) $PM_1$ prediction

## 5. Sensitivity Analysis of Temporal Resolution

To evaluate the impact of temporal aggregation on model performance, we conducted a sensitivity analysis. The original 10-second data were aggregated into time windows ranging from 20 seconds to 10 minutes (i.e., 20s, 30s, 1 min, 2 min, ..., 10 min). We evaluated the LightGBM model's performance ($R^2$ and MSE) across these temporal scales. As shown in **Figure S 8**, the model performance initially improves with longer aggregation times, reaching a peak at the 3-minute resolution ($R^2$ = 82.42% for $PM_{2.5}$ and 81.64% for $PM_1$; MSE reaches its minimum). This improvement is primarily because appropriate time averaging effectively filters out instrumental white noise and transient micro-environmental anomalies. However, beyond 3 minutes, the performance gradually declines. While the 3-minute aggregation yields the highest $R^2$, it corresponds to a large spatial span along the monitoring route. This extensive spatial coverage causes severe spatial over-smoothing, masking the hyper-local pollution hotspots that our mobile monitoring campaign aimed to capture. Therefore, to preserve the ultra-high spatiotemporal resolution necessary for street-level exposure assessment, we retained the 10-second and 1-minute resolutions for the main analyses in this study.

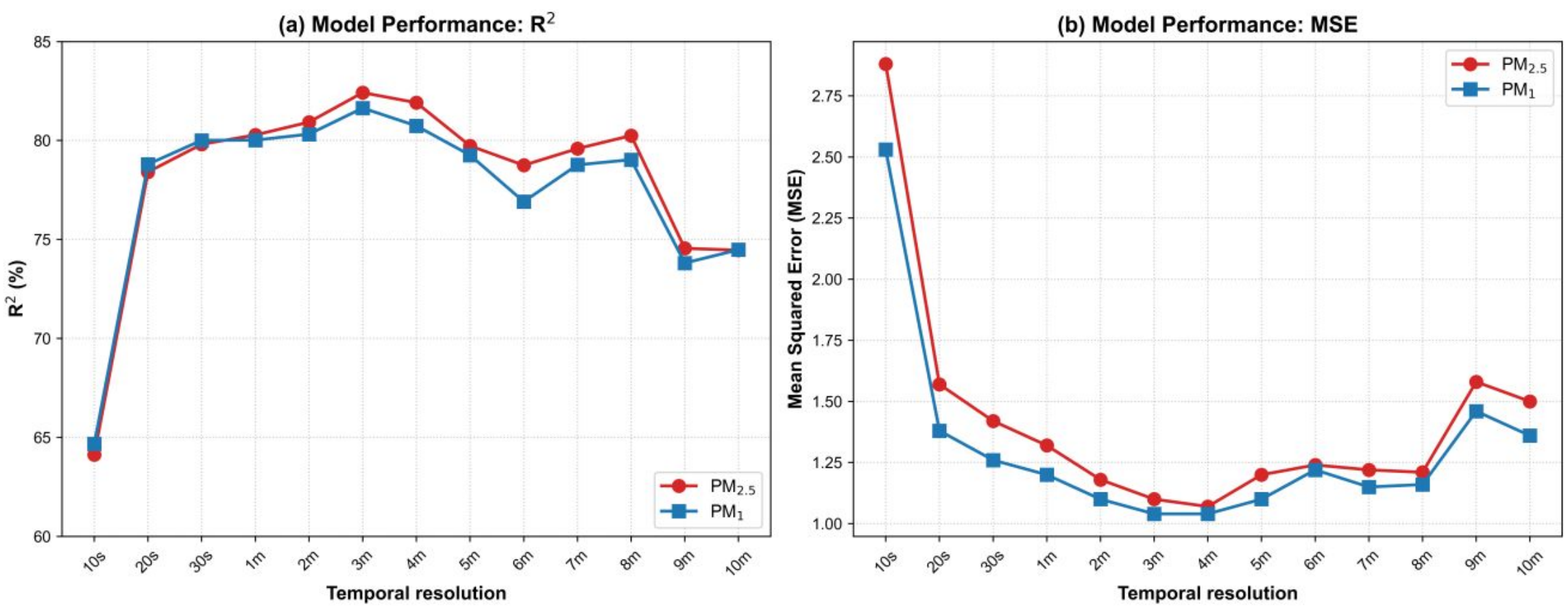


Figure S 8 Sensitivity of the model performance to different temporal resolutions for predicting: (a) $R^2$; (b) MSE.

## Reference


(1) Brattich, E.; Bracci, A.; Zappi, A.; Morozzi, P.; Di Sabatino, S.; Porcù, F.; Di Nicola, F.; Tositti, L. How to Get the Best from Low-Cost Particulate Matter Sensors: Guidelines and Practical Recommendations. *Sensors* **2020**, *20* (11), 3073. https://doi.org/10.3390/s20113073.

(2) Verma, D.; Jana, A.; Ramamritham, K. Machine-Based Understanding of Manually Collected Visual and Auditory Datasets for Urban Perception Studies. *Landsc. Urban Plan.* **2019**, *190*, 103604. https://doi.org/10.1016/j.landurbplan.2019.103604.

(3) Verma, D.; Jana, A.; Ramamritham, K. Predicting Human Perception of the Urban Environment in a Spatiotemporal Urban Setting Using Locally Acquired Street View Images and Audio Clips. *Build. Environ.* **2020**, *186*, 107340. https://doi.org/10.1016/j.buildenv.2020.107340.

(4) Gemmeke, J. F.; Ellis, D. P.; Freedman, D.; Jansen, A.; Lawrence, W.; Moore, R. C.; Plakal, M.; Ritter, M. Audio Set: An Ontology and Human-Labeled Dataset for Audio Events. In *2017 IEEE international conference on acoustics, speech and signal processing (ICASSP)*; IEEE, 2017; pp 776–780.

(5) Gong, Y.; Chung, Y.-A.; Glass, J. AST: Audio Spectrogram Transformer. arXiv July 8, 2021. http://arxiv.org/abs/2104.01778 (accessed 2024-05-29).

(6) Zhou, S.; Lin, R. Spatial-Temporal Heterogeneity of Air Pollution: The Relationship between Built Environment and on-Road PM2.5 at Micro Scale. *Transp. Res. Part Transp. Environ.* **2019**, *76*, 305–322. https://doi.org/10.1016/j.trd.2019.09.004.

(7) Yu, X.; Ma, J.; Jiang, F. Revealing the Impacts of the Built Environment Factors on Pedestrian-Weighted Air Pollutant Concentration Using Automated and Interpretable Machine Learning. *J. Environ. Manage.* **2025**, *387*, 125850. https://doi.org/10.1016/j.jenvman.2025.125850.

(8) Zhang, A.; Xia, C.; Li, W. Exploring the Effects of 3D Urban Form on Urban Air Quality: Evidence from Fifteen Megacities in China. *Sustain. Cities Soc.* **2022**, *78*, 103649. https://doi.org/10.1016/j.scs.2021.103649.

(9) Wang, C.; Wu, Q.; Weimer, M.; Zhu, E. FLAML: A Fast and Lightweight Automl Library. *Proc.*

*Mach. Learn. Syst.* **2021**, *3*, 434–447.

(*Cited literature are also listed in the* ***References*** *of the main text)*
